# The Neisseria gonorrhoeae Accessory Genome and Its Association with the Core Genome and Antimicrobial Resistance

**DOI:** 10.1128/spectrum.02654-21

**Published:** 2022-05-23

**Authors:** Jolinda de Korne-Elenbaas, Sylvia M. Bruisten, Alje P. van Dam, Martin C. J. Maiden, Odile B. Harrison

**Affiliations:** a Public Health Laboratory, Department of Infectious Diseases, Public Health Service of Amsterdam, Amsterdam, the Netherlands; b Amsterdam UMC, University of Amsterdam, Department of Medical Microbiology, Amsterdam Institute for Infection and Immunity (AI&II), Academic Medical Center, Amsterdam, the Netherlands; c Amsterdam UMC, University of Amsterdam, Amsterdam Institute for Infection and Immunity (AI&II), Amsterdam, the Netherlands; d Department of Zoology, Sir William Dunn School of Pathology, University of Oxford, Oxford, United Kingdom; Peking University People's Hospital

**Keywords:** *Neisseria gonorrhoeae*, pangenome, core genome, accessory genome, population biology, antimicrobial resistance

## Abstract

The bacterial accessory genome provides the genetic flexibility needed to facilitate environment and host adaptation. In Neisseria gonorrhoeae, known accessory elements include plasmids which can transfer and mediate antimicrobial resistance (AMR); however, chromosomal accessory genes could also play a role in AMR. Here, the gonococcal accessory genome was characterized using gene-by-gene approaches and its association with the core genome and AMR were assessed. The gonococcal accessory gene pool consisted of 247 genes, which were mainly genes located on large mobile genetic elements, phage associated genes, or genes encoding putative secretion systems. Accessory elements showed similar synteny across genomes, indicating either a predisposition for particular genomic locations or ancestral inheritance that are conserved during strain expansion. Significant associations were found between the prevalence of accessory elements and core genome multi-locus sequence types (cgMLST), consistent with a structured gonococcal population despite frequent horizontal gene transfer (HGT). Increased prevalence of putative DNA exchange regulators was significantly associated with AMR, which included a putative secretion system, methyltransferases and a toxin-antitoxin system. Although frequent HGT results in high genetic diversity in the gonococcus, we found that this is mediated by a small gene pool. In fact, a highly organized genome composition was identified with a strong association between the accessory and core genome. Increased prevalence of DNA exchange regulators in antimicrobial resistant isolates suggests that genetic material exchange plays a role in the development or maintenance of AMR. These findings enhance our understanding of gonococcal genome architecture and have important implications for gonococcal population biology.

**IMPORTANCE** The emergence of antimicrobial resistance (AMR) against third generation cephalosporins in Neisseria gonorrhoeae is a major public health concern, as these are antibiotics of last resort for the effective treatment of gonorrhea. Although the resistance mechanisms against this class of antibiotics have not been entirely resolved, resistance against other classes of antibiotics, such as tetracyclines, is known to be mediated through plasmids, which are known gonococcal extra-chromosomal accessory elements. A complete assessment of the chromosomal accessory genome content and its role in AMR has not yet been undertaken. Here, we comprehensively characterize the gonococcal accessory genome to better understand genome architecture as well as the evolution and mechanisms of AMR in this species.

## INTRODUCTION

Neisseria gonorrhoeae, the gonococcus, is a Gram negative, oxidase positive diplococcus belonging to the *Neisseriaceae* family. It is arguably the only pathogen in the genus *Neisseria* colonizing the *tractus urogenitalis* as an obligate pathogen, with its close relative Neisseria meningitidis more frequently described as an opportunistic or accidental pathogen (1). Gonococcal urogenital colonization in men invariably results in disease pathology, with untreated gonococcal infection potentially leading to epididymitis. Urogenital infections in women often stay asymptomatic and therefore untreated, potentially causing pelvic inflammatory disease (2, 3). Effective and prompt treatment of infection is therefore essential; however, this is threatened by the emergence of multidrug resistance in the gonococcus which is further exacerbated by an increasing prevalence of N. gonorrhoeae infections globally (3). This is particularly concerning given the emergence of reduced susceptibility against currently used third generation cephalosporins because these are the antibiotics of last resort. In addition, asymptomatic infections of the pharynx, rectum, or female urogenital tract facilitate ongoing transmission of N. gonorrhoeae strains with reduced susceptibility. Understanding antimicrobial resistance (AMR) mechanisms in the gonococcus and tracking the spread of resistant strains is therefore important to limit infections globally.

In N. gonorrhoeae, AMR is both plasmid and chromosomally mediated, depending on the class of antibiotics. Genetic determinants for fluoroquinolone and macrolide resistance have high predictive value with regard to susceptibility phenotype (4). In contrast, determinants mediating resistance against third generation cephalosporins are less predictive and still contain unknown “factor X” genetic determinants, indicating that mechanisms of resistance have not been entirely resolved (4). As a result, to fully expose the mechanisms of AMR, a more conclusive understanding of genome content across the gonococcal population is needed to comprehensively evaluate the evolution and mechanisms of AMR in this species.

For most bacterial species, genome content is not fixed but rather consists of a flexible gene pool, known as the pangenome (5). The pangenome can be further divided into: (i) the core genome, comprised of genes that are present in all isolates of a species and which are essential for survival, and (ii) the accessory genome, consisting of genes present in only a subset of the population and providing the genetic flexibility to facilitate niche adaptation and phenotypic variation (6, 7). The accessory genome can be shaped through evolutionary and environmental selective pressure resulting in gene acquisition or loss (8). Antimicrobial selection, for example, plays an important role in shaping bacterial genome content and in structuring bacterial populations, with bacteria acquiring resistance determinants possessing a selective advantage over susceptible ones when exposed to antibiotics. This development has been observed in several bacterial species, including the gonococcus (9).

Studies assessing the gonococcal core genome have identified the presence of a residual clonal structure that retains gene synteny despite frequent horizontal gene transfer (HGT), with some lineages found to be more resistant to antimicrobials than others (10). In addition, the distribution of mobile genetic elements including plasmids, which form part of the accessory genome, was found to be associated with the gonococcal core genome (11). Although plasmids are important mobile genetic elements that transfer and mediate AMR, chromosomal accessory genes could also play a role. However, the accessory genome content as well as its implications for phenotypic characteristics in N. gonorrhoeae are largely unknown. Here, we characterized the gonococcal accessory genome and examined its association with the core genome and AMR. Our findings reveal that the gonococcal gene pool was smaller than expected with respect to the high genetic diversity known to exist in this species. In addition, genome composition was highly organized with conserved gene synteny and a close association between accessory genome content and the core genome. These findings enhance our understanding of the gonococcal genome architecture and have important implications for gonococcal population biology.

## RESULTS

### Quality of sequence data and isolate characteristics.

For accessory genome characterization, genome sequence data from 765 publicly available isolates were chosen, dating from 1979 to 2019 and originating from 42 countries (Table S1). Draft genomes were available for 764 of 765 isolates with a median number of 123 contigs, and a complete genome was available for one isolate. Median GC content was 52.5%, the total median assembly length was 2,125,586 bp and a median of 1,632 alleles (range 1,460 to 1,652) was designated. Isolates belonged to 289 different multilocus sequence types (MLST) of which 204 were represented by a single isolate and 24 by ≥5 isolates. Sixty-five isolates belonged to MLST ST-7827, being the predominant MLST in the data set. There were 199 different core genome MLST (cgMLST) groups assigned, when using the threshold of 300 or fewer locus differences to differentiate between cgMLST groups (Ng_cgc_300), of which 148 were represented by a single isolate and 23 by ≥5 isolates. The cgMLST_300 group 3 predominated in the data set with 88 isolates belonging to it. These isolates dated from 2004 to 2019 and mainly originated from the Netherlands (64/88, 79%) (Table S1). The largest proportion of isolates in cgMLST_300 group 3 belonged to MLST ST-1901 (43/88, 49%), similar to the predominant group of isolates in PubMLST which also belong to cgMLST_300 group 3 and MLST ST-1901 (1,140/8,013, 14%, April 2021).

**FIG 1:**
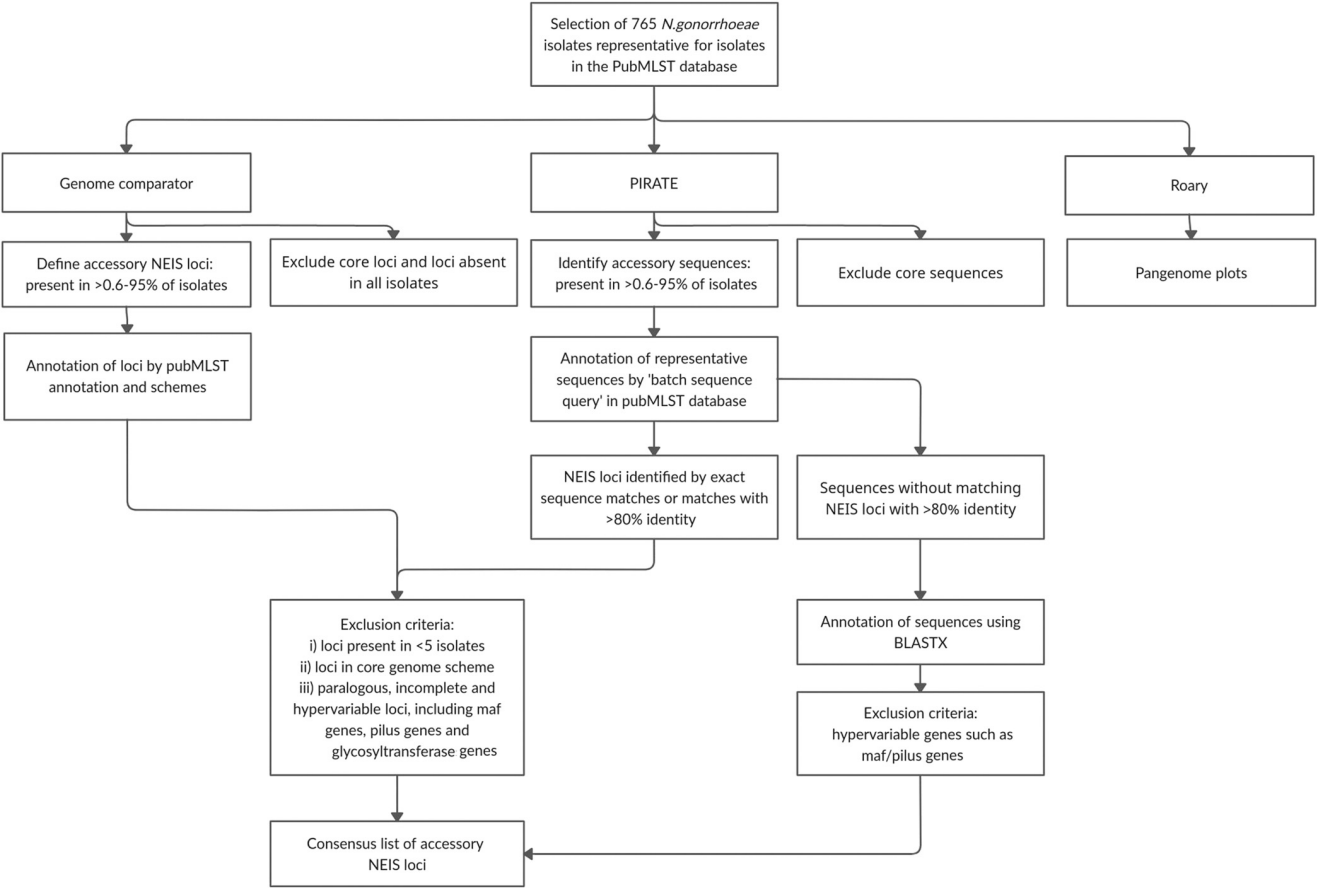
Workflow for the characterization of the accessory genome in N. gonorrhoeae. Genome Comparator, PIRATE, and Roary software were used on a data set of 765 isolates.

To determine to what extent the subset of 765 isolates was representative for the whole database of 8,013 isolates publicly available in PubMLST (accessed April 2021), a minimum spanning tree was generated including these isolates. Locations of the 765 isolates were visualized, revealing a distribution throughout the tree consistent with the selection of a heterogeneous data set that was representative of all publicly available N. gonorrhoeae isolates (Fig. S1). The workflow of characterization of the accessory genome using these 765 isolates is visualized in Fig. 1.

### The gonococcal gene pool has a limited size.

**FIG 2:**
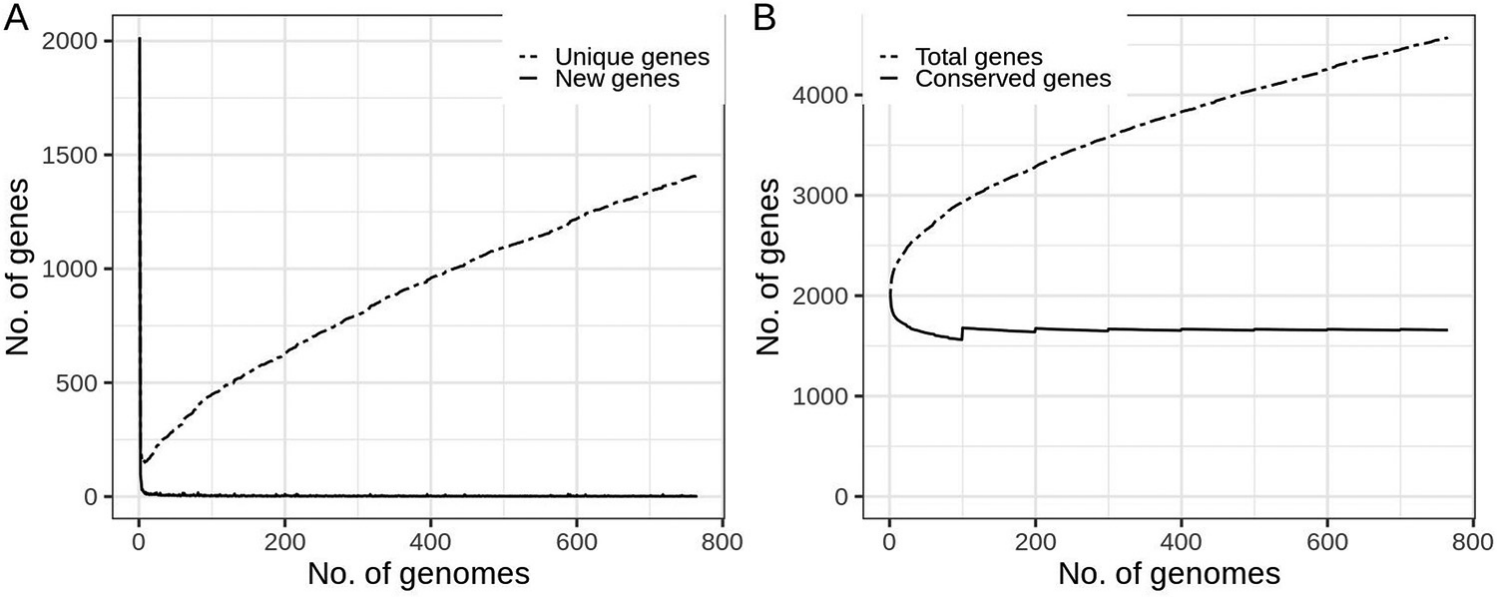
Roary pangenome plots demonstrating relationships between numbers of genes identified and numbers of genomes used. The numbers of new (A) and conserved (B) genes saturized with less than 20 genomes. The numbers of unique (A) and total (B) genes were positively correlated with the number of genomes included in the analysis, as shown by a constant increase of identified genes when the number of genomes increased.

Roary pangenome plots were examined to assess the relationships between numbers of genes identified and numbers of genomes used. These plots showed that saturation in the number of new (Fig. 2A) and conserved genes (Fig. 2B) identified was reached after including fewer than 20 genomes, consistent with a restricted gonococcal pangenome size. As a feature, Roary splits variants of the same gene into multiple unique genes where these have less than 95% similarity. Therefore, the number of unique (Fig. 2A) and total genes (Fig. 2B) identified correlated with the number of gene variants defined, which in turn increased as more genomes were examined. This indicates that, in the gonococcus, diversity within genes increases proportionally as more genomes are examined, whereas diversity in genome content saturizes with less than 20 genomes. This also showed that the subset of 765 isolates was sufficient to capture the full extent of the gonococcal pangenome.

### The gonococcal accessory genome contained 247 loci.

All 2,859 NEIS loci defined in PubMLST.org/neisseria were compared in the 765 isolates using Genome Comparator. This showed that 573 loci were absent in all 765 isolates, 24 loci were present in fewer than five isolates (prevalence: <0.6%) and 292 were accessory (prevalence: 0.6% to 95%). Filtering resulted in a final list of 196 accessory loci based on Genome Comparator analyses. PIRATE identified 3,189 distinct gene sequences of which 795 were present in fewer than five isolates and 623 were accessory. Querying of these 623 gene sequences in PubMLST showed that there were duplicate sequences of single NEIS loci, falsely identified as separate genes by PIRATE. As a result, the 623 gene sequences matched to 209 unique NEIS loci with exact matches, of which 173 had also been identified as accessory using Genome Comparator. Eight NEIS loci were identified as accessory by PIRATE only; however, these loci were determined as core by Genome Comparator and were therefore excluded from further analyses. Genome Comparator identified 23 accessory loci not identified by PIRATE. For 93 gene sequences identified as accessory by PIRATE, no matches to NEIS loci with >80% identity were found. Sequence annotation and filtering resulted in a list of 82 unique accessory genes identified by PIRATE only, of which 58 were added as NEIS loci to PubMLST (NEIS3177 to 3235, except NEIS3201) and of which 51 were confirmed to be accessory after screening isolates for these loci with the Gene Presence tool available in PubMLST. Twenty-four sequences were not added because these were either predicted to be non-functional or encoded transposases. Taken together, the consensus list contained 247 unique accessory NEIS loci which are provided in Table S2, together with prevalences and functions. A detailed workflow showing all steps taken to obtain the final list of 247 accessory NEIS loci is provided in Fig. S2.

### The gonococcal accessory genome mainly consists of large mobile genetic elements.

Functional annotation of identified accessory genes showed that most of genes were located on large mobile genetic elements, namely: 67 genes on the gonococcal genomic island (GGI) (27%), 51 on the conjugative plasmid (21%), and eight on the beta-lactamase plasmid (3%). Aside from the mobile genetic elements, the major constituent of the accessory genome consisted of 31 hypothetical genes (12.5%), 30 genes encoding the VirB type IV secretion system (VirB T4SS) (12%), and 34 phage associated genes (13.5%) (Table 1). Phage-associated genes were found both as part of prophages (16/34 genes on Nf1, Nf4 and phage island X) or individual genes scattered over the chromosome. One group of seven genes (NEIS0080 to 0089), annotated as hypothetical or genes encoding protein export proteins in PubMLST, formed a genomic island with a constant synteny across genomes. Detailed BLAST annotation showed that this island encodes a putative secretion system. Other accessory genes encode methyltransferases, toxin-antitoxins or proteins involved in DNA transcription and replication (Table 1).

**TABLE 1:** Functional annotations of 247 identified accessory genes in N. gonorrhoeae

Functional category	Subcategory	No. of accessory genes	%
Mobile genetic elements	Gonococcal genomic island (GGI)	67	(27%)
	Conjugative plasmid	51	(21%)
	Beta-lactamase plasmid	8	(3%)
VirB Type IV secretion system		30	(12%)
Putative secretion system		7	(3%)
Methyltransferases (outside genomic islands)		6	(2.5%)
Phage associated genes	Nf1 phage island	6	(2.5%)
	Nf4 phage island	3	(1%)
	Phage island X associated	7	(3%)
	(Putative) phage associated	18	(7%)
Genetic information processing	DNA transcription	3	(1%)
	DNA replication	1	(0.5%)
Toxin-antitoxin system		2	(1%)
TonB- dependent receptors		3	(1%)
Other functions	Alcohol dehydrogenase	1	(0.5%)
	Putative peptidase	1	(0.5%)
	Membrane proteins	1	(0.5%)
	TspB virulence factor	1	(0.5%)
Hypothetical genes		31	(12.5%)
Total no. of accessory genes		247	100%

### Chromosomal accessory elements show similar synteny across genomes.

**FIG 3:**
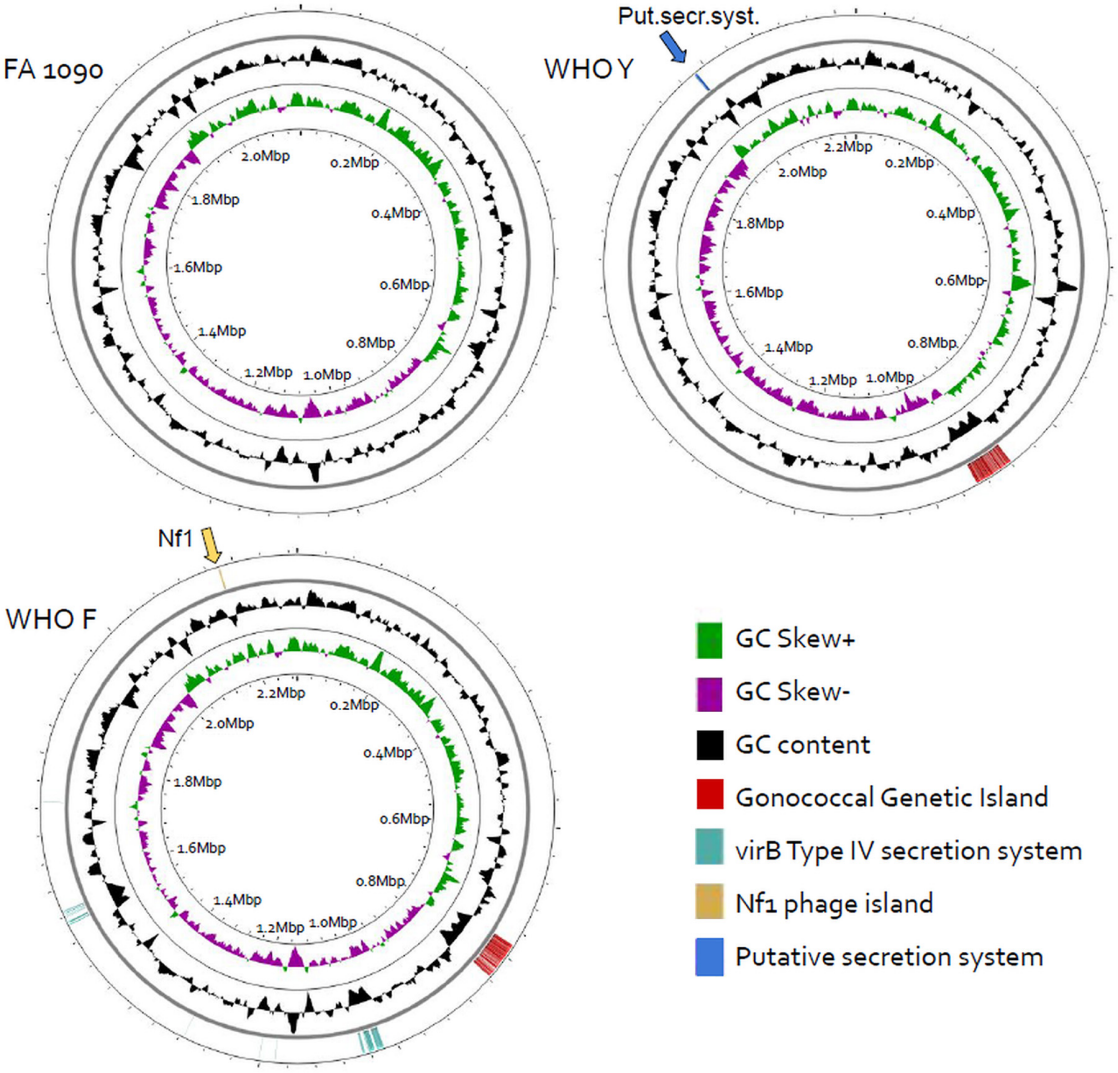
Genomic locations of chromosomal accessory elements belonging to the gonococcal accessory genome. Locations were visualized on circular genomes of well-known reference strains. Reference strain FA1090 lacks all accessory elements and its chromosomal accessory genome is the smallest of three. Accessory elements were identified in WHO-Y and WHO-F, of which WHO-F has the largest accessory genome. From inner to outer circle: GC skew, GC content, reading frame with annotated genes.

The main chromosomal accessory elements were visualized using reference genomes FA1090, WHO-Y, and WHO-F (Fig. 3). WHO-F has the largest chromosomal accessory genome, including the GGI, VirB T4SS, and Nf1 phage island. WHO-Y has the GGI and the putative secretion system while FA1090 has the smallest chromosomal accessory genome and lacks all major elements. Interestingly, the accessory elements displayed similar synteny which indicates either predisposition for these elements in these locations or ancestral inheritance, conserved during strain expansion.

### The accessory genome is highly structured and associated with the core genome.

**FIG 4:**
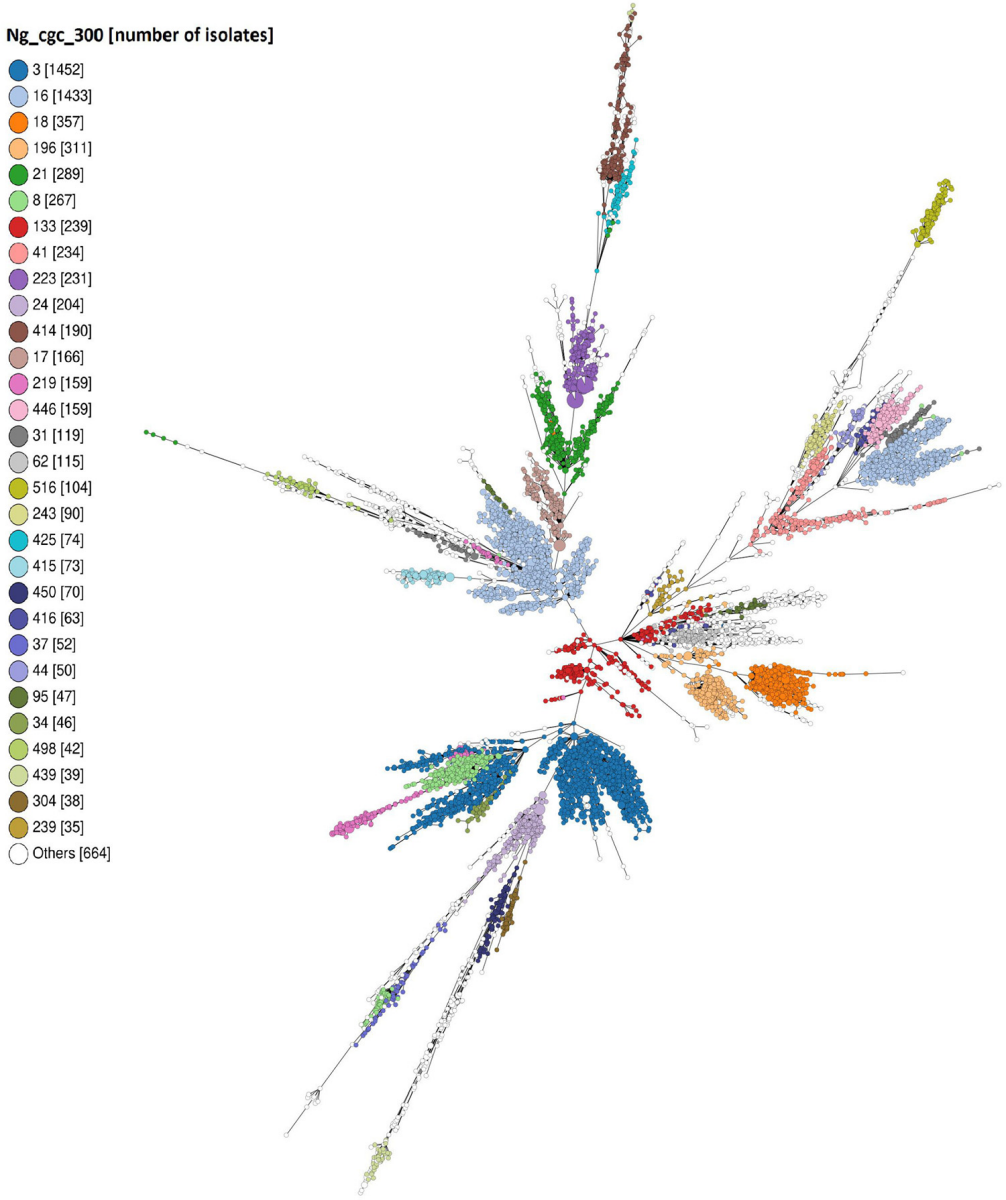
Minimum-spanning tree based on the N. gonorrhoeae accessory genome MLST typing scheme created in PubMLST. All 8,013 publicly available N. gonorrhoeae isolates were included in the tree. Colors represent core genome MLST groups using the 300 or fewer locus threshold (Ng_cgc_300). Clustering of colors indicated that the gonococcal accessory- and core genomes are strongly associated, confirming that the gonococcal genome is highly structured. Two separate clusters could be identified for the largest core genome MLST groups 3 and 16. The presence of these two clusters indicates divergence in both core and accessory genome content and a requirement for more stringent locus thresholds to be used to delineate these clusters. The tree was created with GrapeTree and nodes were positioned through dynamic rendering, meaning that branch lengths are not scaled. Legend shows the core genome MLST group (300 or fewer locus threshold).

After characterization of the accessory genome in the subset of 765 isolates, associations between the accessory and core genome and/or AMR were assessed using all 8,013 genomes available in PubMLST (accessed April 2021). The accessory genome typing scheme (agMLST v1.0) was created in PubMLST including all identified accessory loci. Based on this scheme, a minimum-spanning tree was generated including all 8,013 isolates. Nodes were colored by cgMLST group and clustering of colors demonstrated that there was a strong relationship between accessory and core genomes (Fig. 4).

Among the 8,013 isolates, the GGI was present in 69% (5,524/8,013), the conjugative plasmid in 27% (2,174/8,013), and the beta-lactamase plasmid in 11% (887/8,013). Associations between cgMLST and accessory elements were examined and results of the main cgMLST groups with >35 isolates are shown in Table 2. When comparing the prevalence of accessory elements within a cgMLST group to the prevalence among all isolates, the GGI, conjugative plasmid, beta-lactamase plasmid, and the genes encoding the putative secretion system were either significantly more prevalent or significantly less prevalent in most of the cgMLST groups (*P-*value range: <0.05 - <0.0001; Table 2 and Table S3). The Nf1 phage island was significantly more prevalent in isolates belonging to cgMLSTs 18, 415, and 304 (*P-*values <0.0001), compared with the distribution among all isolates. Isolates belonging to cgMLSTs 3, 16, 18, and 196 showed little variation in accessory genome content, which is striking given the large numbers of isolates in these groups. Isolates belonging to cgMLSTs 21, 8, and 133 had a more variable accessory genome content with almost half of the isolates possessing the GGI or beta-lactamase plasmid (Table 2). The strong association found between accessory and core genome is consistent with a structured gonococcal population. This also indicated ancestral inheritance of accessory elements in certain gonococcal lineages which are conserved during lineage expansion with limited spread to other lineages.

**TABLE 2:** Prevalence of accessory elements and significance of associations with cgMLST groups (Ng_cgc_300)

CgMLST	No. of isolates	Gonococcal genetic island		Conjugative plasmid		Betalactamase plasmid		VirB T4SS		Nf1 phage island		Putative secretion	
		Prevalence	Sign.^*a*^	Prevalence	Sign.^*a*^	Prevalence	Sign.^*a*^	Prevalence	Sign.^*a*^	Prevalence	Sign.^*a*^	Prevalence	Sign.^*a*^
3	1452	99%	****^c^	0%	****^b^	1%	****^b^	0%	*^b^	0%	****^b^	97%	****^c^
16	1433	20%	****^b^	0%	****^b^	0%	****^b^	0%	*^b^	0%	****^b^	92%	****^c^
18	357	90%	****^c^	1%	****^b^	2%	****^b^	0%		15%	****^b^	0%	****^b^
196	311	100%	****^c^	4%	****^b^	0%	****^b^	0%		0%	**^b^	0%	****^b^
21	289	41%	****^b^	93%	****^c^	77%	****^c^	0%		0%	**^b^	0%	****^b^
8	267	52%	****^b^	0%	****^b^	0%	****^b^	0%		0%	*^b^	95%	****^b^
133	239	59%		100%	****^b^	31%	****^c^	0%		0%		93%	****^c^
41	234	99%	****^c^	100%	****^c^	48%	****^c^	0%		0%		0%	****^b^
223	231	99%	****^c^	1%	****^b^	0%	****^b^	0%		0%		0%	****^b^
24	204	100%	****^c^	2%	****^b^	0%	****^b^	0%		0%		0%	****^b^
414	190	100%	****^c^	7%	****^b^	1%	****^b^	0%		0%		0%	****^b^
17	166	81%		1%	****^b^	1%	***^b^	0%		0%		95%	****^c^
219	159	9%	****^b^	1%	****^b^	4%		0%		0%		0%	****^b^
446	159	100%	****^c^	100%	****^c^	1%	***^b^	0%		0%		1%	****^b^
31	119	54%		12%	*^b^	1%	**^b^	0%		0%		74%	****^c^
62	115	12%	****^b^	100%	****^c^	47%	****^c^	0%		0%		1%	****^b^
516	104	100%	****^c^	0%	****^b^	34%	****^c^	0%		0%		0%	****^b^
243	90	100%	****^c^	76%	****^c^	2%		0%		0%		0%	****^b^
425	74	100%	****^c^	49%	*^c^	3%		0%		1%		1%	****^b^
415	73	100%	****^c^	1%	****^b^	0%		0%		100%	****^c^	0%	****^b^
450	70	97%	****^c^	100%	****^c^	1%		1%		0%		0%	****^b^
416	63	8%	****^b^	100%	****^c^	33%	***^c^	0%		0%		0%	****^b^
37	52	100%	****^c^	88%	****^c^	79%	****^c^	0%		0%		2%	****^b^
44	50	100%	****^c^	14%		0%		0%		0%		0%	****^b^
95	47	32%	****^b^	66%	****^c^	4%		0%		0%		98%	****^c^
34	46	98%	***^c^	0%	***^b^	0%		0%		0%		89%	****^c^
498	42	100%	****^c^	100%	****^b^	17%		0%		0%		0%	****^b^
439	39	5%	****^b^	90%	****^c^	0%		0%		0%		0%	****^b^
304	38	97%	**^c^	100%	****^c^	3%		5%		89%	****^c^	0%	****^b^
239	35	9%	****^b^	100%	****^c^	83%	****^c^	0%		0%		91%	****^c^
Other	675												
No cgMLST	601												
Overall	8013	69%		27%		11%		0.7%		3.6%		48%	

a Adjusted *p*-values were calculated by multiplying the original *p*-value by the total number of core genome groups tested (*n* = 250). *P < 0.05; **P < 0.01; ***P < 0.001; ****P < 0.0001.

b Significantly less prevalent in cgMLST group compared to distribution among all isolates.

c Significantly more prevalent in cgMLST group compared to distribution among all isolates.

### Accessory genes involved in DNA uptake and excretion are associated with AMR.

Associations between the accessory genome and resistance against penicillin, tetracycline, ciprofloxacin, ceftriaxone, cefixime, and azithromycin were assessed. As expected, the beta-lactamase plasmid was significantly more prevalent among penicillin- (*P* < 0.0001) or tetracycline- (*P* < 0.0001) resistant isolates, compared with susceptible isolates. However, this was also found for ciprofloxacin-resistant isolates (*P* < 0.0001). Interestingly, the genes encoding the putative secretion system (NEIS0080 to 0089), all methyltransferases (NEIS1193 to 1194, NEIS1311, NEIS2691 to 2692) except one (NEIS3182), a DNA transcription protein (NEIS3184) and a toxin-antitoxin system (NEIS3188 to NEIS3232) were significantly more prevalent among isolates resistant to penicillin, tetracycline, ciprofloxacin, cefixime, or azithromycin (*P-*value range: <0.05 to <0.0001; Table 3 and Table S4 and S5). The low number of ceftriaxone resistant isolates (*n* = 22) hampered identification of significant associations, although genes encoding toxin-antitoxin systems were significantly more prevalent in ceftriaxone resistant isolates (*P* < 0.05) and trends of increasing prevalence were seen for the other genes. The two hypothetical genes NEIS0364 to 0365, which always occur as a pair, were also significantly more prevalent in isolates resistant to all antibiotics except ceftriaxone (*P*-values <0.0001). BLAST annotation showed that NEIS0365 encodes a predicted competence protein which interacts with incoming DNA. Altogether, these findings indicated increased prevalence of genes encoding DNA uptake and excretion proteins in resistant isolates, independent of the class of antibiotic.

**TABLE 3:** Prevalence of accessory elements in isolates with available phenotypic data and the significance of associations

			Gonococcal genetic island		Conjugative plasmid		Betalactamase plasmid		virB T4SS		Nf1 phage island		Putative secretion system	
Susceptibility^*a*^	MIC	No. of isolates	Prevalence	Sign.^*b*^	Prevalence	Sign.^*b*^	Prevalence	Sign.^*b*^	Prevalence	Sign.^*b*^	Prevalence	Sign.^*b*^	Prevalence	Sign.^*b*^
PEN S	≤0.06	117	89%	****^c^	32%	****	0%	**** ^*d*^	7%	****^c^	7%		16%	**** ^*d*^
PEN I	>0.06-1.0	1896	64%		23%		2%		1%		3%		52%	
PEN R	>1.0	1074	74%		33%		33%		0%		3%		53%	
TET S	≤0.5	774	74%	***^c^	23%	* ^*d*^	8%	**** ^*d*^	2%	***^c^	4%		37%	**** ^*d*^
TET R	>0.5	2143	66%		28%		14%		1%		3%		57%	
CIP S	≤0.03	2098	65%	** ^*d*^	26%		6%	**** ^*d*^	2%	****^c^	3%	**^c^	42%	****^d^
CIP R	>0.06	2316	70%		29%		16%		0%		2%		56%	
CRO S	≤0.125	4868	67%		26%		11%		1%		2%		51%	
CRO R	>0.125	22	91%		14%		0%		0%		0%		77%	
CFX S	≤0.125	4,004	66%	**** ^*d*^	29%	****^c^	12%	****^c^	1%		3%	****^c^	47%	****^d^
CFX R	>0.125	490	85%		2%		1%		0%		0%		93%	
AZI S	<1.0	3547	71%	****^c^	29%	****^c^	13%	****^c^	1%		3%		45%	**** ^*d*^
AZI R	≥1.0	898	60%		8%		2%		0%		1%		75%	

a PEN = penicillin; TET = tetracycline; CIP = ciprofloxacin; CRO = ceftriaxone; CFX = cefixime; AZI = azithromycin; S = susceptible; I = intermediate; R = resistant.

b Adjusted *P*-values were calculated by multiplying the original *P*-value by the number of accessory elements tested (*n* = 6). *P < 0.05; **P < 0.01; ***P < 0.001; ****P < 0.0001.

c Significantly more prevalent among susceptible isolates.

d Significantly more prevalent among resistant isolates.

## DISCUSSION

This study used a robust and detailed gene-by-gene approach to characterize the gonococcal accessory genome. Since the introduction of the pangenome concept in bacterial research, multiple computational tools have been developed to examine pangenome content (12). Tools such as Roary and PIRATE are based on clustering algorithms which use gene similarity thresholds to identify gene families. These thresholds can be difficult to define as a consequence of major differences in diversity among genes. This often results in over-splitting of divergent alleles of the same gene into multiple clusters or over-clustering of related gene families. PIRATE tries to improve this by measuring sequence diversity within the data set and correcting gene clusters based on this (13). However, the output obtained here still contained far more gene clusters than genes identified in PubMLST, indicating over-splitting. This was evidenced by querying the identified PIRATE sequences in PubMLST, with multiple sequences matching the same NEIS locus. This therefore shows the necessity for careful examination of raw outputs resulting from pangenome tools for undertaking research on a gene level. Furthermore, gene-by-gene analysis combined with in-depth knowledge of the bacterial species is important to translate pangenome outputs from theoretical to functional genes.

The bacterial accessory genome provides genetic flexibility, facilitating host and environmental adaptation. In the gonococcus, major accessory elements include plasmids, mediating resistance against beta-lactams and tetracycline. The content of the chromosomal accessory genome and its implications in population biology and AMR are largely unknown. Due to frequent intraspecies HGT, high genetic diversity is found in gonococci exemplified by the challenges encountered in vaccine development and rapidity with which AMR develops. Such genetic diversity was also expected in the accessory genome, however, our results showed that a small gene pool constituted the accessory genome with the GGI and plasmids being the main accessory elements. Characterization of the chromosomal gonococcal accessory genome revealed that it was highly organized consistent with retention of gene synteny despite frequent HGT and that it was associated with the core genome. Therefore, the main driver of allelic variation in the gonococcal population is the high rate of genetic exchange, whereas gene acquisition occurs to a much lesser extent (14). This is evidenced by the rapid saturation in new genes identified once a small number of genomes were examined, and the increase in gene diversity seen as more genomes were included (Fig. 2). Recombination being predominant to gene gain and loss has also been observed for Chlamydia trachomatis, suggesting a typical genome constitution for sexually transmitted pathogens (15). The highly structured genome organization and small gene pool size are indicative of a founder effect resulting from an ancestral gonococcus first transitioning to the urogenital tract, and resulting in a niche switch and a change in genome content that has been selected for and preserved. This could explain why the *Neisseria* genus consists mostly of commensal and symbiotic species residing in the human nasopharynx, with the gonococcus being unusual in that it is principally found in the male and female urogenital tracts (1).

The limited gonococcal gene pool is possibly a consequence of the gonococcus being for the most part an obligate pathogen. Compared with opportunistic pathogens that are highly adaptable to diverse environments through their extensive accessory genomes, obligate pathogens often have limited pangenomes, a consequence of reductive evolution due to host restriction (5, 16–18). Gene sharing is also more prevalent among species that share the same habitat, and the gene diversity in the habitat determines the size and diversity of the species’ gene pool (6, 7). Species existing in diverse communities often have an open and highly variable pangenome, while “niche specialists” that exist in stable, less variable, and host-restricted environments have much smaller and more conserved pangenomes (8). The gonococcus is such a host-restricted pathogen, predominantly inhabiting the human urogenital tract and despite the male and female urogenital tract being different environments, the gonococcus can be considered a niche specialist. Pharyngeal gonococcal infections nonetheless occur, which will facilitate interspecies genetic exchange between N. gonorrhoeae, N. meningitidis, and commensal *Neisseria* inhabiting the nasopharynx (19). These pharyngeal infections are considered to be the main driver for uptake of antibiotic resistance mutations from other *Neisseria* species, facilitated by the high prevalence of these infections in key populations (20). Genetic exchange has also been shown to occur from gonococci to urogenital associated meningococci, of which increasing cases have been reported over the last decade (21, 22). This shows the potential for genetic exchange between these two *Neisseria* species (23). Further studies assessing the prevalence of gonococci and meningococci co-existing in the same environment may determine the likelihood of increased HGT between them.

The gonococcal accessory and core genomes were associated, indicating ancestral inheritance of accessory elements in certain gonococcal lineages which are conserved during lineage expansion with limited spread to other lineages. This is consistent with a previous finding showing that plasmid distribution was lineage-associated in the gonococcus (11). Similar structuring of bacterial populations, with strong associations between lineage and accessory genome content, has been found in other bacterial species, including Escherichia coli (24). This indicates that bacterial lineages are structured by both core and accessory genome content. Genomic elements, such as transposons or insertion sequences, play key roles in intra- and interchromosomal rearrangements (25). For example, GGI uptake is regulated by the GGI insertion sequence and recombination in this region leads to GGI loss (26). Further examination of these elements might inform whether lineage specific prevalence of accessory elements is associated with particular insertion sequences. Examination of these sequences, which often contain repetitive regions, is challenging using current short-read sequencing techniques. Such studies are foreseeable in the near future with the advent of cheaper and more accessible long-read sequencing techniques.

Significant associations were found between increased prevalence of DNA uptake and exchange regulators and AMR, suggesting a role for genetic exchange in the development or maintenance of AMR. This also supports the hypothesis of certain genetic backgrounds being predisposed for resistance, as in the gonococcus resistance is highly associated with the core genome and specific lineages are more resistant than others (10). Domenech et al. suggested reducing the spread of AMR by inhibiting bacterial competence (27). Important to note is that the prevalence indicated in this study is only at the gene level, whereas upregulation of DNA exchange and uptake should be confirmed by functional examination at the gene expression level.

### Conclusions.

Despite frequent HGT being responsible for high genetic diversity in the gonococcus, the gonococcal gene pool was found to be small with the accessory genome consisting mainly of the GGI and plasmids. In addition, the gonococcal accessory genome was associated with the core genome, consistent with the ordered population structure previously identified. These data indicate that recombination predominantly drives allelic variation and to a much lesser extent gene acquisition or loss. Increased prevalence of genes encoding DNA exchange regulators in resistant gonococcal isolates suggests that genetic material exchange may play a role in the development or maintenance of AMR. Understanding the role of such mechanisms will be important to limit the spread of AMR in the gonococcus. The findings presented here enhance our understanding of gonococcal genome architecture and have important implications for gonococcal population biology.

## MATERIALS AND METHODS

### Isolate collection and whole genome sequence data.

The PubMLST.org/neisseria database contained 8,013 publicly available N. gonorrhoeae isolates with whole genome sequence data available (accessed April 2021). A subset of 765 N. gonorrhoeae isolates was chosen that was representative of the total N. gonorrhoeae genomes found in PubMLST. This data set of 765 isolates included (i) 380 isolates from a Dutch surveillance study containing many different MLSTs (28) and (ii) 385 isolates chosen to be representative of all MLSTs, core genome groups, and geographical origins available in PubMLST (Table S1). The extent to which this data set was representative of the diversity found in all 8,013 publicly available isolates was assessed through the use of a minimum spanning tree generated using the PubMLST plugin GrapeTree (29). Using this tool, all 8,013 isolates were compared with the N. gonorrhoeae cgMLST v1.0 core genome scheme (10), and the distribution of the subset of 765 isolates in the tree was assessed. Tree nodes were positioned through dynamic rendering, meaning that branch lengths are not scaled. Genome assembly metrics were obtained for all isolates to validate genome quality. In addition, species identity was confirmed using the ribosomal MLST species identification tool which also controls for species contamination (pubmlst.org/rmlst).

### Identification of accessory loci.

The PubMLST *Neisseria* database allows genomes across the *Neisseria* genus to be annotated and as a result contains gene definitions belonging to multiple *Neisseria* species. A total of 2,859 loci have been defined in PubMLST that are representative of the *Neisseria* gene pool. Using the Genome Comparator tool available in PubMLST, these loci were compared in the 765 isolates (30). Alleles defined as “incomplete” or “undefined” were considered present because this might be due to hypervariability of the gene or to assembly errors. Core genes were defined as loci found in 95% to 100% of genomes, whereas accessory genes were defined as loci present in 0.6% to 95% of genomes (loci present in <5 genomes were excluded, equaling 0.6%). Loci considered to be core were excluded from further analyses.

A sequence-based approach was also used to identify loci that may not have been defined in PubMLST. Identification and classification of orthologous genes within the 765 isolates was done with PIRATE v1.0.4 using genomes previously annotated with Prokka v1.13-v1.14.5 (13, 31). Default settings were used. Pangenome plots were produced with Roary v3.13.0 using default settings except paralogue splitting was disabled (32). These plots were used to assess the relationship between genes identified and the number of genomes added. Similar criteria as used for Genome Comparator were employed to define core and accessory genes identified by PIRATE. Representative sequences of the identified accessory gene families were queried in PubMLST to find corresponding NEIS loci. Sequences that matched only partially to a NEIS locus were queried against a custom database including all 1,547,524 alleles (accessed June 2021) of all 2,859 NEIS loci using BLASTN with a minimum sequence identity of 80%. Sequences that did not match any NEIS allele with >80% identity were queried against the N. gonorrhoeae (taxid: 485) BLASTX database to get (predicted) functional annotations (33). Novel sequences were then defined in PubMLST using the NEIS nomenclature. BLAST searches were performed to screen isolates for these novel loci using the Gene Presence plugin with the following thresholds: minimum sequence identity 95% and minimum sequence alignment 95%. Finally, a N. gonorrhoeae typing scheme was defined consisting of the N. gonorrhoeae: (i) accessory genome (agMLST); (ii) core genome (cgMLST); and (iii) pangenome (pgMLST) (Workflow visualized in Fig. 1).

### Filtering of identified accessory loci.

NEIS loci assigned as accessory by Genome Comparator and PIRATE were filtered as follows: (i) exclusion of loci that were present in <5 isolates; (ii) exclusion of loci that were part of the PubMLST N. gonorrhoeae cgMLST v1.0 scheme of PubMLST; these are most likely erroneously in the accessory genome due to incompleteness of the genes in draft genome data; and (iii) exclusion of paralogous, incomplete and hypervariable loci, including those belonging to the multiple adhesin family (*maf*) island, pilus and glycosyltransferase schemes of PubMLST. The paralogous nature and hypervariability of these genes leads to uncertainty when estimating prevalence in draft genome data. After filtering, lists from Genome Comparator and PIRATE were combined to produce a consensus list of accessory NEIS loci (Fig. 1).

### Functional characterization and distribution of accessory loci.

Identified accessory loci were classified into functional groups using annotations defined in PubMLST. Locations of all accessory loci were visualized using the reference genomes: WHO-F (PubMLST ID: 62968; GenBank accession number: LT591897.1), WHO-Y (PubMLST ID: 88866; GenBank accession number: LT592161.1), and FA1090 (PubMLST ID: 2855; GenBank accession number: AE004969.1) in CGviewer (34). Loci annotated as “hypothetical” but located on *maf*-islands were excluded from the list of accessory genes as these are paralogous. Duplicate sequences of the same loci were identified by performing sequence queries in PubMLST. The distribution of accessory loci across isolates was assessed using the Gene Presence plugin in PubMLST with the following thresholds: minimum sequence identity 95% and minimum sequence alignment 95%. To identify genomic islands or operons, co-location of loci was assessed based on gene distribution patterns. A genomic island or operon was considered present when >75% of its genes were identified and these were in adjacent locations. Phage associated genes NEIS0027 to 0031 and NEIS2451 were assigned to the Nf1 phage island as defined by Al Suwayyid et al. (35).

### Association between core- and accessory genome.

An agMLST scheme was created in PubMLST, including all identified accessory NEIS loci. The association between core and accessory genome was assessed including all 8,013 isolates, through the use of a minimum spanning tree generated with the GrapeTree tool. The tree was built based on the agMLST scheme with the “rescan undesignated loci” option enabled. Nodes were colored by cgMLST group using the 300 or fewer locus threshold (Ng_cgc_300) and positioned through dynamic rendering, meaning that branch lengths are not scaled.

### Accessory genome distribution in 8,013 publicly available isolates.

Using the agMLST scheme, prevalence and distribution of the accessory NEIS loci were assessed in all 8,013 isolates in PubMLST, using the Gene Presence plugin as described above. Prevalence of accessory elements were determined for cgMLST groups that contained >35 isolates and using the 300 or fewer locus threshold (Ng_cgc_300).

### Statistics.

Fisher’s exact tests were performed to identify significant associations between the presence of accessory elements and cgMLST group as well as the association with resistance against penicillin, tetracycline, ciprofloxacin, ceftriaxone, cefixime, and azithromycin. When testing for associations with resistance, only isolates that had phenotypic data for that specific antibiotic were included in analyses. Phenotypic data was categorized as “susceptible” or “resistant” (or “intermediate” for penicillin) according to EUCAST breakpoints v11.0 (36). The Bonferroni correction method was used to correct for multiple testing and *P*-values were adjusted accordingly. Analyses were performed in R v3.6.3.

### Data availability.

The data sets supporting the conclusions of this article are available in the PubMLST Neisseria database repository (https://pubmlst.org/organisms/neisseria-spp). PubMLST IDs can be found in Table S1.
