# Supplementary material for: The Neisseria gonorrhoeae Accessory Genome and Its Association with the Core Genome and Antimicrobial Resistance
Source: Microbiol Spectr. 2022 May 23;10(3):e02654-21. doi: 10.1128/spectrum.02654-21 (PMC9241924; doi:10.1128/spectrum.02654-21)
Supplement: SUPPLEMENTAL FILE 1 — Supplemental material. Download spectrum.02654-21-s001.pdf, PDF file, 3.7 MB [file spectrum.02654-21-s001.pdf]

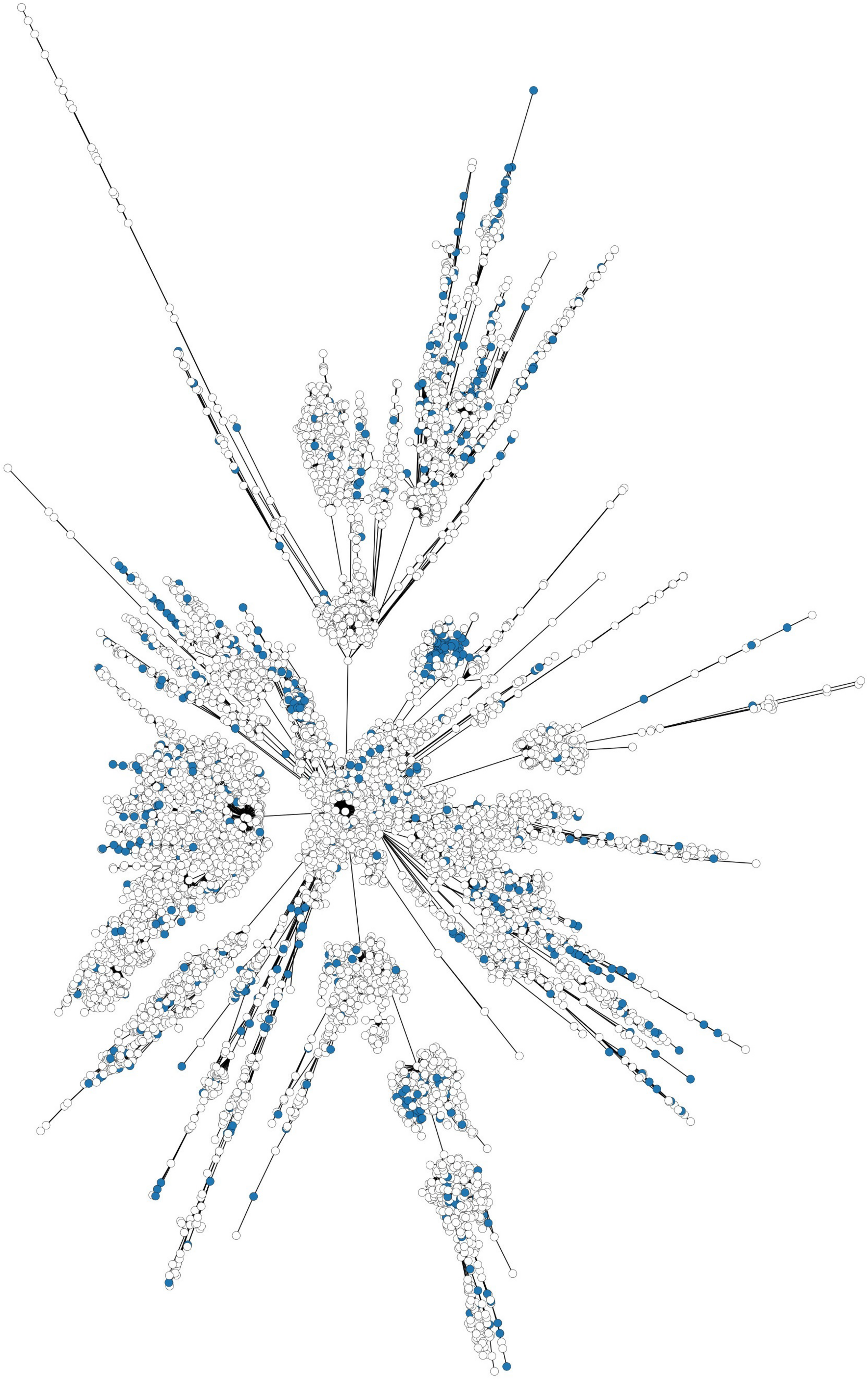

Figure S1. Minimum-spanning tree based on *N. gonorrhoeae* cgMLST v1.0 typing scheme including all 8,013 *Neisseria gonorrhoeae* isolates available in PubMLST. The 765 isolates included in the representative subset of isolates used for accessory genome characterization are colored in blue. The blue nodes are distributed throughout the tree consistent with heterogeneous, non-biased sampling. The tree was created with GrapeTree and nodes were positioned through dynamic rendering, meaning that branch lenghts are not scaled.

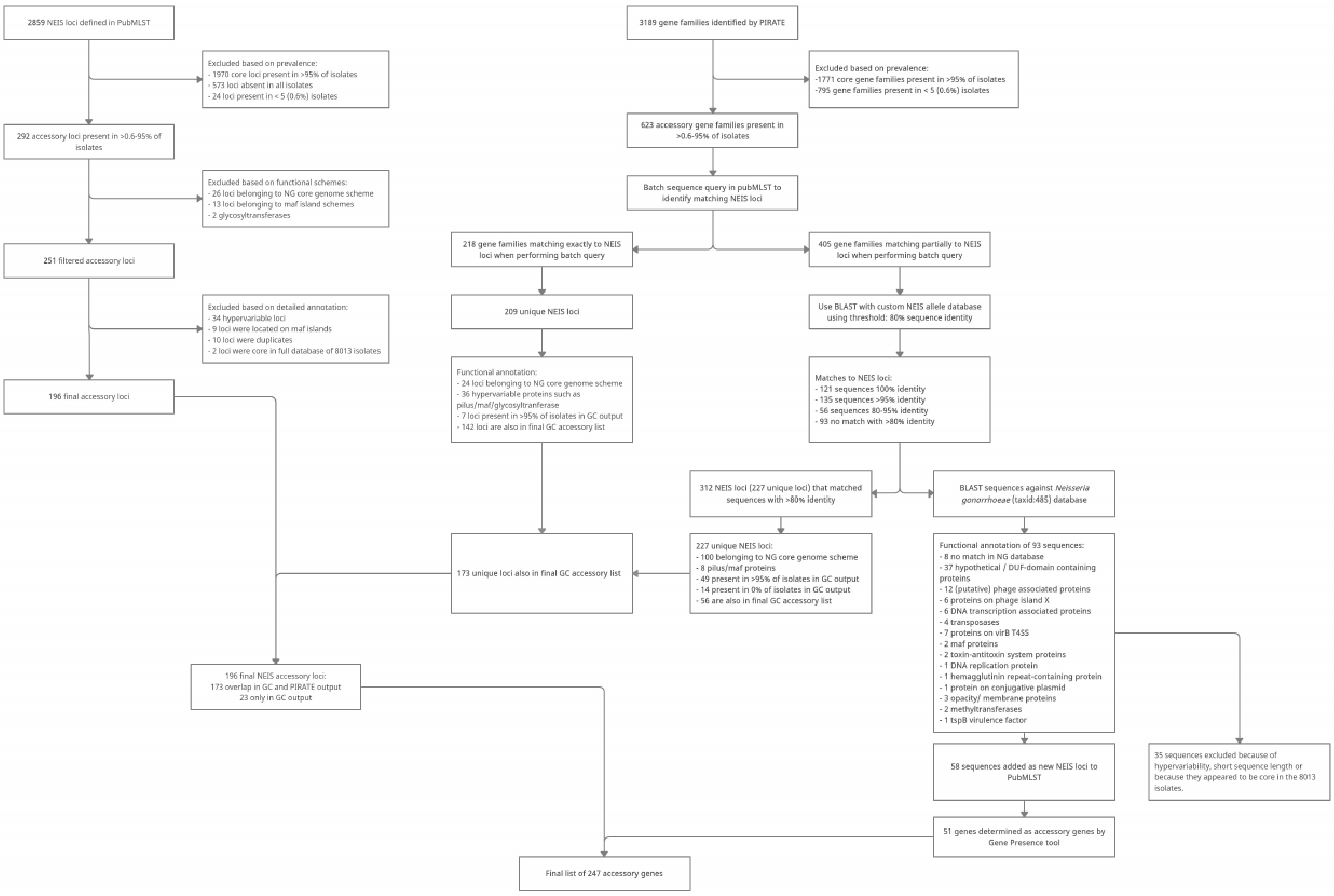

Figure S2. Detailed workflow and results of each filtering step during accessory genome identification.

Table S1. Isolate characteristics

PubMLST ID	Country	Continent	Year of isolation	MLST	NG-MAST v2.0	Core genome group
27235	USA	North America	2009	1583	766	17
27270	USA	North America	2009	6722	19326	22
27302	USA	North America	2009	10315	1103	3
27327	USA	North America	2009	1901	225	18
27353	USA	North America	2009	1579	286	3
27364	USA	North America	2009	1582	4424	32
27467	USA	North America	2009	8110	2265	3
27471	USA	North America	2009	10316	7468	420
31458	Norway	Europe	2009	1901	3149	3
31463	Germany	Europe	2008	1901	3698	18
31464	Greece	Europe	2008	11173	2272	416
31470	Belarus	Europe	2009	11174	2873	425
31477	Belarus	Europe	2009	8126	1929	16
31480	Estonia	Europe	2010	1588	211	21
31482	Estonia	Europe	2010	1594	7485	414
31487	Belarus	Europe	2010	1595	7436	427
31496	Slovenia	Europe	2010	10241	5616	429
31499	Estonia	Europe	2011	11175	1241	428
31510	Slovenia	Europe	2011	7823	5405	133
31512	Estonia	Europe	2012	6726	19335	431
31522	China	Asia	1998	1580	1085	56
31524	China	Asia	1998	7360	16120	18
31525	China [Hong Kong]	Asia	1998	11077	16139	432
31526	Indonesia	Asia	1998	6715	1691	433
31527	Malaysia	Asia	1998	1582	147	416
31529	Philippines	Asia	1998	1583	1083	389
31533	Thailand	Asia	1998	7371	10855	306
31535	Thailand	Asia	1998	1583	16141	307
31544	India	Asia	2009	1600	6074	436
31545	India	Asia	2010	11180	6082	71
31547	India	Asia	2010	11181	6073	437
31548	Bhutan	Asia	2010	8776	6064	390
31553	Cuba	North America	1998	11182	16131	75
31554	Cuba	North America	1998	11183	16115	76
31556	Cuba	North America	1998	11184	16134	308
31557	Jamaica	North America	1998	11185	545	78
31559	Brazil	South America	2008	1583	3791	440
31561	Ecuador	South America	2008	11186	3771	81
31566	USA	North America	1999	8127	3305	16

31568	UK	Europe	1986	1931	6207	84
31572	UK	Europe	1990	1906	8	442
31573	UK	Europe	1993	8152	170	443
31574	UK	Europe	1993	11181	175	311
31575	UK	Europe	1993	10899	176	411
31577	UK	Europe	1993	1920	178	444
31578	Denmark	Europe	1998	1579	21	24
31581	Greece	Europe	1998	1902	495	42
31582	Norway	Europe	1998	1591	16133	445
31584	Poland	Europe	1998	1601	16116	426
31586	Russia	Asia	1998	11188	807	90
31587	Russia	Asia	1998	9903	19336	90
31590	Spain	Europe	1998	1595	8	442
31593	Spain	Europe	1999	1580	64	16
31594	The Netherlands	Europe	1999	12078	277	90
31598	Russia	Asia	2004	6810	1532	447
31604	Denmark	Europe	2008	1893	3766	31
31608	Poland	Europe	2008	11189	3798	448
31612	Turkey	Asia	2008	11177	1993	430
31627	Estonia	Europe	2009	8776	7483	449
31631	Slovenia	Europe	2009	1901	225	18
31633	Belarus	Europe	2010	10631	7476	3
31637	Poland	Europe	2010	1898	5421	21
31639	Poland	Europe	2010	7363	8376	315
31646	Poland	Europe	2011	9363	8390	16
31651	Slovenia	Europe	2011	8131	8658	42
31654	Estonia	Europe	2012	1931	7487	303
31655	Estonia	Europe	2012	1892	5185	425
31662	Thailand	Asia	1979	1920	172	451
31663	Australia	Oceania	1992	7367	3304	95
31665	Australia	Oceania	1996	1599	904	101
31666	Australia	Oceania	1997	1890	270	452
31669	China	Asia	1998	11193	16138	57
31670	Indonesia	Asia	1998	7365	16121	317
31671	Malaysia	Asia	1998	7367	8804	83
31672	Philippines	Asia	1998	11194	16124	104
31673	Philippines	Asia	1998	10634	16126	60
31674	Thailand	Asia	1998	11195	16140	105
31675	Thailand	Asia	1998	1583	3427	106
31676	Thailand	Asia	1998	11196	16142	107
31677	Thailand	Asia	1998	8143	3032	416
31678	Thailand	Asia	1998	11197	16143	108

31686	USA	North America	2009	10311	19337	418
31688	Thailand	Asia	1999	1582	16119	454
31689	Thailand	Asia	1999	1601	3031	392
31690	Thailand	Asia	1999	7369	16135	393
31701	India	Asia	2007	11199	6348	455
31702	China	Asia	2008	10314	3777	3
31703	China	Asia	2008	6715	3755	133
31705	Indonesia	Asia	2008	1891	201	394
31707	Philippines	Asia	2008	11200	3754	456
31708	Thailand	Asia	2008	11201	3759	21
31709	India	Asia	2009	12079	19338	458
31711	India	Asia	2009	8134	6091	37
31715	Pakistan	Asia	2009	1903	368	37
31716	Pakistan	Asia	2009	11074	6095	37
31721	India	Asia	2010	1927	6085	460
31722	India	Asia	2010	6813	6086	461
31723	India	Asia	2010	11191	368	124
31724	India	Asia	2010	11204	6057	125
31726	Bhutan	Asia	2010	11205	6061	462
31735	Pakistan	Asia	2011	1599	6319	127
31740	Japan	Asia	2001	1596	8132	8
31745	Germany	Europe	1998	11207	12970	409
31755	Australia	Oceania	2004	7365	1257	64
31756	Japan	Asia	2001	7363	4091	130
31758	Australia	Oceania	2007	1903	621	64
31760	Vietnam	Asia	2008	9364	3770	21
31764	Australia	Oceania	2001	1583	556	132
31766	Thailand	Asia	2008	11210	2233	416
31768	Russia	Asia	2004	8160	758	16
31769	Thailand	Asia	2008	1925	3761	416
31779	Thailand	Asia	2008	8145	3796	320
31787	Italy	Europe	1998	1584	685	41
31796	Thailand	Asia	2008	8112	1458	135
31797	New Zealand	Oceania	2004	8122	210	446
31798	France	Europe	1998	12080	132	465
31799	Russia	Asia	2004	6810	1525	466
31801	Germany	Europe	2008	10622	3760	467
31802	UK [Scotland]	Europe	2005	8148	210	468
31805	Russia	Asia	2004	1905	1517	469
31812	Russia	Asia	2004	11212	1538	142
31814	India	Asia	2008	8775	6058	459
31815	Indonesia	Asia	1998	1931	16122	143

31817	The Netherlands	Europe	1998	11188	16151	144
31946	Pakistan	Asia	2011	11228	6079	146
31952	Vietnam	Asia	2011	10313	7724	263
31953	Vietnam	Asia	2011	1600	7154	148
31954	Vietnam	Asia	2011	11230	7741	133
31955	Vietnam	Asia	2011	11231	4676	149
31968	South Africa	Africa	1991	11234	182	473
31969	South Africa	Africa	1991	10899	183	474
31971	Cape Verde	Africa	1998	1583	758	475
31972	Cape Verde	Africa	1998	1931	16146	155
31974	The Gambia	Africa	1998	8139	3187	156
31976	Morocco	Africa	1998	1583	1247	90
31977	Morocco	Africa	1998	11235	16147	157
31978	Tanzania	Africa	1998	12082	16148	476
31979	Uganda	Africa	1999	8142	16127	477
31981	The Gambia	Africa	1999	1585	231	44
31982	Guinea	Africa	1999	12083	16128	478
31983	Morocco	Africa	1999	7363	16129	479
31986	Guinea-Bissau	Africa	2007	1603	3187	156
31994	Guinea-Bissau	Africa	2007	11236	783	480
31996	Guinea-Bissau	Africa	2007	11240	3179	481
31997	Guinea-Bissau	Africa	2007	11241	3377	481
31998	Guinea-Bissau	Africa	2007	8123	3180	327
31999	Guinea-Bissau	Africa	2007	8161	5302	166
32002	Guinea-Bissau	Africa	2007	11242	3183	481
32010	Guinea-Bissau	Africa	2007	11244	3376	155
32011	Guinea-Bissau	Africa	2007	12084	3182	482
32074	Saudi Arabia	Asia	1998	7363	16150	184
32076	UK	Europe	2002	1892	261	489
37029	USA	North America	2007	11411	19361	435
37100	USA	North America	2012	11412	19364	31
37108	USA	North America	2012	12007	19103	446
37122	USA	North America	2006	10935	19369	78
37124	USA	North America	2007	1579	19371	18
37125	USA	North America	2007	11413	1318	198
37142	USA	North America	2011	8149	9103	506
37143	USA	North America	2011	8136	5256	416
37161	USA	North America	2011	1582	19375	200
37167	USA	North America	2011	12087	10639	31
37171	USA	North America	2011	11414	5276	507
37177	USA	North America	2011	12088	7117	3
37186	USA	North America	2012	12089	19378	415

37188	USA	North America	2012	12090	19134	509
37190	USA	North America	2012	10316	5618	507
37191	USA	North America	2012	6962	8223	421
37196	USA	North America	2012	1889	19380	31
37197	USA	North America	2012	11415	19381	204
37198	USA	North America	2012	1920	19114	418
37203	USA	North America	2012	11184	752	335
37205	USA	North America	2012	7828	8420	510
37251	USA	North America	2006	1590	19391	505
37265	USA	North America	2007	12091	5	24
37307	USA	North America	2005	12092	19397	44
37327	USA	North America	2005	1590	19400	512
37357	USA	North America	2007	1932	19096	336
37377	USA	North America	2008	10241	19120	3
37390	USA	North America	2009	8130	758	25
37396	USA	North America	2010	11418	19110	41
37404	USA	North America	2011	1600	8503	196
37405	USA	North America	2011	11419	9246	416
37413	USA	North America	2011	7828	1614	211
37457	USA	North America	2011	7363	7574	196
37492	USA	North America	2013	8149	8247	513
37536	USA	North America	2013	7363	13479	196
37547	USA	North America	2013	11423	10941	17
45019	Australia	Oceania	2011	7363	10103	521
45022	Australia	Oceania	2011	8145	436	234
45031	Australia	Oceania	2011	1901	225	18
45032	Australia	Oceania	2013	12041	10125	524
45037	Australia	Oceania	2012	7360	1407	3
45042	Australia	Oceania	2013	1890	8842	21
45050	Australia	Oceania	2012	1924	10105	227
45051	Australia	Oceania	2012	12045	758	520
45057	Australia	Oceania	2012	12042	9716	522
45061	Australia	Oceania	2013	7363	10117	525
45062	Australia	Oceania	2013	12044	19435	526
45063	Australia	Oceania	2013	12046	8063	520
45069	Australia	Oceania	2013	10317	5268	41
45071	Australia	Oceania	2013	8156	7268	17
45075	Australia	Oceania	2013	7359	4186	223
45330	Australia	Oceania	2013	8149	10120	527
46853	Norway	Europe	2015	12459	11745	241
46858	Norway	Europe	2015	12460	18917	416
46868	Norway	Europe	2016	12461	8561	62

46875	Norway	Europe	2016	13923	19245	425
46876	Norway	Europe	2016	12000	12433	196
46887	Norway	Europe	2016	9900	18926	133
46889	Norway	Europe	2015	14333	292	446
46897	Norway	Europe	2015	8776	9983	236
46902	Norway	Europe	2015	11985	568	133
46908	Norway	Europe	2016	9362	2992	16
46910	Norway	Europe	2016	8774	18930	529
46923	Norway	Europe	2016	13924	2992	16
46924	Norway	Europe	2016	14334	18933	18
46926	Norway	Europe	2016	8136	18934	530
46939	Norway	Europe	2016	14332	18937	96
46949	Norway	Europe	2016	10316	12496	251
46957	Norway	Europe	2016	14335	18941	96
46960	Norway	Europe	2016	11750	18940	254
46962	Norway	Europe	2016	1579	18944	344
47751	UK [England]	Europe	2014	7822	4995	133
47758	UK [England]	Europe	2014	11420	3935	16
47776	UK [England]	Europe	2014	1583	217	538
48622	UK [England]	Europe	2009	11990	51	414
48644	UK [England]	Europe	2009	1931	19089	350
48650	UK [England]	Europe	2008	7826	2487	223
48655	UK [England]	Europe	2010	7363	2400	196
48701	UK [England]	Europe	2007	1892	7344	540
48708	UK [England]	Europe	2007	1901	19478	18
48727	UK [England]	Europe	2010	8775		274
48741	UK [England]	Europe	2010	10622	1730	528
48748	UK [England]	Europe	2010	8163	2	439
48758	UK [England]	Europe	2009	11417	2992	16
48769	UK [England]	Europe	2005	8114	4	44
48844	UK [England]	Europe	2004	8148	210	468
48851	UK [England]	Europe	2004	1587	1186	208
48884	UK [England]	Europe	2004	1904	748	510
48891	UK [England]	Europe	2005	12529	766	17
48902	UK [England]	Europe	2004	8392	377	486
48947	UK [England]	Europe	2004	6960	709	541
49169	UK [England]	Europe	2009	12533	19485	16
49188	UK [England]	Europe	2007	12508	3	439
49194	UK [England]	Europe	2007	1918	785	415
49201	UK [England]	Europe	2009	1600	19486	16
50479	Norway	Europe	2016	14336	8953	3
50488	Norway	Europe	2016	14337	18951	239

50491	Norway	Europe	2016	11249	18952	21
51116	Norway	Europe	2016	13590	9158	3
51661	Norway	Europe	2017	11367	377	542
51675	Norway	Europe	2017	13252	15257	96
51685	Norway	Europe	2017	13266	11421	62
51688	Norway	Europe	2017	7827	15188	21
51699	Norway	Europe	2017	11999	995	16
51705	Norway	Europe	2017	13142	1691	62
51707	Norway	Europe	2017	8135	10676	425
51711	Norway	Europe	2017	13292	9208	16
51742	Norway	Europe	2017	11516	5793	516
51744	Norway	Europe	2017	8143	5624	62
51754	Norway	Europe	2017	11986	13487	17
51763	Norway	Europe	2017	8780	5446	21
51766	Norway	Europe	2017	8784	5447	416
51770	Norway	Europe	2017	13309	3110	281
51773	Norway	Europe	2017	1929	18976	543
51801	Norway	Europe	2017	11431	18710	544
51802	Norway	Europe	2017	11365	18979	21
51813	Norway	Europe	2017	12463	16450	249
51819	Norway	Europe	2017	8776	18983	21
51824	Norway	Europe	2017	6716	17436	3
51897	Norway	Europe	2017	13334	14299	17
52154	Norway	Europe	2017	7360	14292	346
52156	Norway	Europe	2017	11189	18970	280
52162	Norway	Europe	2017	11191	18690	96
53452	Norway	Europe	2017	13347	14764	243
53458	Norway	Europe	2017	14339	12302	16
53465	Norway	Europe	2017	10214	18993	133
53466	Norway	Europe	2017	1599	645	243
53561	Norway	Europe	2017	11431	18999	545
53563	Norway	Europe	2017	8130	19000	546
53579	Norway	Europe	2017	1893	14386	498
53586	Norway	Europe	2017	13143	2318	219
53614	Norway	Europe	2017	11249	19006	236
53625	Norway	Europe	2017	14342	18288	21
53626	Norway	Europe	2017	11968	5624	62
53645	Norway	Europe	2017	8123	19010	62
53658	Norway	Europe	2017	1582	5624	62
54284	Brazil	South America	2008	1901	225	18
54298	Brazil	South America	2010	12505	5521	41
54306	Brazil	South America	2010	1596		450

54312	Brazil	South America	2011	1579	19495	95
54314	Brazil	South America	2011	12905	10528	516
54337	Brazil	South America	2013	8133	19502	21
54340	Brazil	South America	2013	13845	340	18
54342	Brazil	South America	2013	1921	338	21
54347	Brazil	South America	2013	8134	2992	16
54362	Brazil	South America	2014	8145	19161	41
54366	Brazil	South America	2014	13550	19506	16
54372	Brazil	South America	2014	11602	12551	3
56128	Norway	Europe	2017	14343	19018	278
56177	Norway	Europe	2017	14345	19025	546
56181	Norway	Europe	2017	14346	9184	196
56184	Norway	Europe	2017	14347	19028	239
56188	Norway	Europe	2017	14348	384	450
56194	Norway	Europe	2017	11864	5049	16
56195	Norway	Europe	2017	1583	14769	133
56198	Norway	Europe	2017	14349	19031	62
56205	Norway	Europe	2017	13409	14769	133
56210	Norway	Europe	2017	1587	13969	239
56218	Norway	Europe	2017	14350	11744	41
56221	Norway	Europe	2017	1894	19035	516
56247	Norway	Europe	2017	1918	8845	509
56259	Norway	Europe	2017	14352	5624	62
56280	Norway	Europe	2017	14354	17194	16
56291	Norway	Europe	2017	14340	13489	17
56293	Norway	Europe	2017	13928	12547	3
56295	Norway	Europe	2017	11208	19045	62
56778	Spain	Europe	2016	7363	13070	8
61062	Norway	Europe	2017	1597	14838	548
61063	Norway	Europe	2017	7827	10386	219
61341	Turkey	Asia	1999	8112	2599	289
61382	Ivory Coast	Africa	2012	8112	9902	162
62968	Canada	North America	1991	10934	3303	441
75910	The Netherlands	Europe	2014	11990	14376	414
75911	The Netherlands	Europe	2014	8156	7268	17
75912	The Netherlands	Europe	2014	1901	19624	3
75913	The Netherlands	Europe	2014	7363	2400	196
75914	The Netherlands	Europe	2014	11864	15192	16
75915	The Netherlands	Europe	2014	11990	14376	414
75916	The Netherlands	Europe	2014	1901	1407	3
75917	The Netherlands	Europe	2014	11864	15192	16
75918	The Netherlands	Europe	2014	9362	2992	16

75919	The Netherlands	Europe	2014	1600	14360	151
75920	The Netherlands	Europe	2014	9363	2992	16
75921	The Netherlands	Europe	2014	1600	14360	151
75922	The Netherlands	Europe	2014	8143	19625	62
75923	The Netherlands	Europe	2014	6726	15726	
75924	The Netherlands	Europe	2014	11990	51	414
75925	The Netherlands	Europe	2014	7827	2318	219
75926	The Netherlands	Europe	2014	11864	6155	16
75927	The Netherlands	Europe	2014	9363	2992	16
75928	The Netherlands	Europe	2014	1600	14360	151
75929	The Netherlands	Europe	2014	8163	2	439
75930	The Netherlands	Europe	2014	8143	5624	62
75931	The Netherlands	Europe	2014	1584	5	41
75932	The Netherlands	Europe	2014	1901	1407	3
75933	The Netherlands	Europe	2014	7363	2400	196
75934	The Netherlands	Europe	2014	9362	2992	16
75935	The Netherlands	Europe	2014	11428	2992	16
75936	The Netherlands	Europe	2014	8112	5560	162
75937	The Netherlands	Europe	2014	7363	7072	8
75938	The Netherlands	Europe	2014	11516	5793	516
75939	The Netherlands	Europe	2014	1901	3935	3
75940	The Netherlands	Europe	2014	11990	14376	414
75941	The Netherlands	Europe	2014	1584	8919	41
75942	The Netherlands	Europe	2014	1901	1407	3
75943	The Netherlands	Europe	2014	1901	1407	3
75944	The Netherlands	Europe	2014	1901	1407	3
75945	The Netherlands	Europe	2014	1588	9276	21
75946	The Netherlands	Europe	2014	9363	2992	16
75947	The Netherlands	Europe	2014	11428	2992	16
75948	The Netherlands	Europe	2014	1901	1407	3
75949	The Netherlands	Europe	2014	7363	9514	196
75950	The Netherlands	Europe	2014	7827	2318	219
75951	The Netherlands	Europe	2014	1901	1407	3
75952	The Netherlands	Europe	2014	9362	2992	16
75953	The Netherlands	Europe	2014	7822	4995	133
75954	The Netherlands	Europe	2014	11428	2992	16
75955	The Netherlands	Europe	2014	11990	14376	414
75956	The Netherlands	Europe	2014	8143	5624	62
75957	The Netherlands	Europe	2014	6720	19626	359
75958	The Netherlands	Europe	2014	12000	10174	196
75959	The Netherlands	Europe	2015	1579	6711	24
75960	The Netherlands	Europe	2015	1893	5444	31

75961	The Netherlands	Europe	2015	7822	14373	133
75962	The Netherlands	Europe	2015	1901	6719	3
75963	The Netherlands	Europe	2015	1584	8919	41
75964	The Netherlands	Europe	2015	1599	11461	243
75965	The Netherlands	Europe	2015	8143	11421	62
75966	The Netherlands	Europe	2015	7363	2400	196
75967	The Netherlands	Europe	2015	1584	4326	41
75968	The Netherlands	Europe	2015	11428	2992	16
75969	The Netherlands	Europe	2015	7363	2400	196
75970	The Netherlands	Europe	2015	7827	2318	219
75971	The Netherlands	Europe	2015	7827	2318	219
75972	The Netherlands	Europe	2015	8156	5441	17
75973	The Netherlands	Europe	2015	7363	2400	196
75974	The Netherlands	Europe	2015	7827	2318	219
75975	The Netherlands	Europe	2015	7827	2318	219
75976	The Netherlands	Europe	2015	11516	5793	516
75977	The Netherlands	Europe	2015	15571	8426	62
75978	The Netherlands	Europe	2015	7823	11210	133
75979	The Netherlands	Europe	2015	11463	2992	16
75980	The Netherlands	Europe	2015	7827	2318	219
75981	The Netherlands	Europe	2015	1599	18208	243
75982	The Netherlands	Europe	2015	7363	6360	196
75983	The Netherlands	Europe	2015	8156	13155	17
75984	The Netherlands	Europe	2015	11428	2992	16
75985	The Netherlands	Europe	2015	7822	5400	133
75986	The Netherlands	Europe	2015	1901	1407	3
75987	The Netherlands	Europe	2015	11428	2992	16
75988	The Netherlands	Europe	2015	11251	11677	549
75989	The Netherlands	Europe	2015	1599	11461	243
75990	The Netherlands	Europe	2015	11990	14376	414
75991	The Netherlands	Europe	2015	1596	384	450
75992	The Netherlands	Europe	2015	7363	17409	8
75993	The Netherlands	Europe	2015	7827	2318	219
75994	The Netherlands	Europe	2016	13143	292	219
75995	The Netherlands	Europe	2016	11428	2992	16
75996	The Netherlands	Europe	2016	11428	3391	16
75997	The Netherlands	Europe	2016	7363	10257	196
75998	The Netherlands	Europe	2016	1579	19627	24
75999	The Netherlands	Europe	2016	8156	16283	17
76000	The Netherlands	Europe	2016	9363	19628	16
76001	The Netherlands	Europe	2016	8156	5441	17
76002	The Netherlands	Europe	2016	7363	13878	8

76003	The Netherlands	Europe	2016	8143	5624	62
76004	The Netherlands	Europe	2016	8163	2	439
76005	The Netherlands	Europe	2016	1599	11461	243
76006	The Netherlands	Europe	2016	9363	9925	16
76007	The Netherlands	Europe	2016	1599	11461	243
76008	The Netherlands	Europe	2016	7363	10789	196
76009	The Netherlands	Europe	2016	8156	5441	17
76010	The Netherlands	Europe	2016	7363	2400	196
76011	The Netherlands	Europe	2016	8156	5441	17
76012	The Netherlands	Europe	2016	1893	19629	31
76013	The Netherlands	Europe	2016	1599	11461	243
76014	The Netherlands	Europe	2016	7827	2318	219
76015	The Netherlands	Europe	2016	9363	8241	16
76016	The Netherlands	Europe	2016	1599	11461	243
76017	The Netherlands	Europe	2016	8156	5441	17
76018	The Netherlands	Europe	2016	11516	19630	516
76019	The Netherlands	Europe	2016	9363	16493	16
76020	The Netherlands	Europe	2016	1901	2958	3
76021	The Netherlands	Europe	2016	1901	2958	3
76022	The Netherlands	Europe	2016	1901	2958	3
76023	The Netherlands	Europe	2016	13292	9208	16
76024	The Netherlands	Europe	2016	1901	2958	3
76025	The Netherlands	Europe	2016	11431	19631	545
76026	The Netherlands	Europe	2016	8156	5441	17
76027	The Netherlands	Europe	2016	7363	15239	196
76028	The Netherlands	Europe	2016	11864	9219	16
76029	The Netherlands	Europe	2016	8163	2	439
76030	The Netherlands	Europe	2016	11864	9219	16
76031	The Netherlands	Europe	2016	7827	2318	219
76032	The Netherlands	Europe	2016	8156	5441	17
76033	The Netherlands	Europe	2016	7827	2318	219
76034	The Netherlands	Europe	2016	7827	2318	219
76035	The Netherlands	Europe	2016	7827	2318	219
76036	The Netherlands	Europe	2017	1599	11461	243
76037	The Netherlands	Europe	2017	11864	15906	16
76038	The Netherlands	Europe	2017	15572	273	360
76039	The Netherlands	Europe	2017	7827	2318	219
76040	The Netherlands	Europe	2017	11428	298	16
76041	The Netherlands	Europe	2017	7827	2318	219
76042	The Netherlands	Europe	2017	11971	15058	62
76043	The Netherlands	Europe	2017	8156	19632	17
76044	The Netherlands	Europe	2017	11428	5582	16

76045	The Netherlands	Europe	2017	8135	387	425
76046	The Netherlands	Europe	2017	1599	11461	243
76047	The Netherlands	Europe	2017	8156	13062	17
76048	The Netherlands	Europe	2017	7827	2318	219
76049	The Netherlands	Europe	2017	11428	5049	16
76050	The Netherlands	Europe	2017	1901	12618	3
76051	The Netherlands	Europe	2017	9363	19633	16
76052	The Netherlands	Europe	2017	7827	10386	219
76053	The Netherlands	Europe	2017	8156	5441	17
76054	The Netherlands	Europe	2017	7827	2318	219
76055	The Netherlands	Europe	2017	7827	10386	219
76056	The Netherlands	Europe	2017	8135	5743	425
76057	The Netherlands	Europe	2017	1596	16972	450
76058	The Netherlands	Europe	2017	9363	12302	16
76059	The Netherlands	Europe	2017	1588	19634	21
76060	The Netherlands	Europe	2017	8135	387	425
76061	The Netherlands	Europe	2017	7359	19635	223
76062	The Netherlands	Europe	2017	13292	9208	16
76063	The Netherlands	Europe	2017	7359	10121	223
76064	The Netherlands	Europe	2017	8156	5441	17
76065	The Netherlands	Europe	2017	10314	16856	3
76066	The Netherlands	Europe	2017	9363	12302	16
76067	The Netherlands	Europe	2018	7822	4995	133
76068	The Netherlands	Europe	2018	10314	16020	3
76069	The Netherlands	Europe	2018	1587	13971	239
76070	The Netherlands	Europe	2018	8156	5441	17
76071	The Netherlands	Europe	2018	8156	5441	17
76072	The Netherlands	Europe	2018	1585	186	44
76073	The Netherlands	Europe	2018	7822	4995	133
76074	The Netherlands	Europe	2018	7827	15710	219
76075	The Netherlands	Europe	2018	10314	16020	3
76076	The Netherlands	Europe	2018	13734	13489	17
76077	The Netherlands	Europe	2018	8163	8110	439
76078	The Netherlands	Europe	2018	11864	18234	16
76079	The Netherlands	Europe	2018	11971	15058	62
76080	The Netherlands	Europe	2018	8156	5441	17
76081	The Netherlands	Europe	2018	11864	18234	16
76082	The Netherlands	Europe	2018	7822	14994	3
76083	The Netherlands	Europe	2018	11971	15058	62
76084	The Netherlands	Europe	2018	1583	15589	
76085	The Netherlands	Europe	2018	10314	19178	3
76086	The Netherlands	Europe	2018	7827	8845	219

76087	The Netherlands	Europe	2018	8156	5441	17
76088	The Netherlands	Europe	2018	11990	51	414
76089	The Netherlands	Europe	2018	8156	5441	17
76090	The Netherlands	Europe	2018	11864	18231	16
76091	The Netherlands	Europe	2018	1583	15589	133
76092	The Netherlands	Europe	2018	11864	5049	16
76093	The Netherlands	Europe	2018	8156	5441	17
76094	The Netherlands	Europe	2018	1583	15589	133
76095	The Netherlands	Europe	2018	1583	14769	133
76096	The Netherlands	Europe	2018	8122	292	446
76097	The Netherlands	Europe	2018	1599	18147	243
76098	The Netherlands	Europe	2018	1583	15589	133
76099	The Netherlands	Europe	2018	8156	5441	17
76100	The Netherlands	Europe	2018	10314	19178	3
76101	The Netherlands	Europe	2018	1583	15589	133
76102	The Netherlands	Europe	2018	8156	5441	17
76103	The Netherlands	Europe	2018	8156	799	17
76104	The Netherlands	Europe	2018	9363	17495	16
76105	The Netherlands	Europe	2018	1587	18131	239
76106	The Netherlands	Europe	2018	9363	12302	16
76107	The Netherlands	Europe	2018	1583	15589	133
76108	The Netherlands	Europe	2018	7822	4654	3
76109	The Netherlands	Europe	2018	15573	17371	16
76110	The Netherlands	Europe	2018	8156	19636	17
76111	The Netherlands	Europe	2018	10314	19188	3
76112	The Netherlands	Europe	2018	7827	14051	219
76113	The Netherlands	Europe	2018	7827	10386	219
76114	The Netherlands	Europe	2018	8156	5441	17
76115	The Netherlands	Europe	2018	7827	10386	219
76116	The Netherlands	Europe	2018	7827	10386	219
76117	The Netherlands	Europe	2018	1583	15589	133
76118	The Netherlands	Europe	2018	9363	3935	16
76119	The Netherlands	Europe	2018	13292	9208	16
76120	The Netherlands	Europe	2018	7827	10386	219
76121	The Netherlands	Europe	2018	1583	15589	133
76122	The Netherlands	Europe	2018	9363	18831	16
76123	The Netherlands	Europe	2018	7827	2318	219
76124	The Netherlands	Europe	2018	1583	15589	133
76125	The Netherlands	Europe	2018	8156	5441	17
76126	The Netherlands	Europe	2018	8156	18362	17
76127	The Netherlands	Europe	2018	7363	8115	196
76128	The Netherlands	Europe	2018	11208	19637	

76129	The Netherlands	Europe	2018	1599	11461	243
76130	The Netherlands	Europe	2018	8135	5743	425
76131	The Netherlands	Europe	2018	8163	19638	439
76132	The Netherlands	Europe	2018	1599	13484	243
76133	The Netherlands	Europe	2019	7822	19552	3
76134	The Netherlands	Europe	2019	8143	5624	62
76135	The Netherlands	Europe	2019	1599	18147	243
76136	The Netherlands	Europe	2019	7822	19559	3
76137	The Netherlands	Europe	2019	11428	4246	16
76138	The Netherlands	Europe	2019	11706	17972	3
76139	The Netherlands	Europe	2019	1599	19639	243
76140	The Netherlands	Europe	2019	7363	9510	196
76141	The Netherlands	Europe	2019	15574	19640	361
76142	The Netherlands	Europe	2019	1599	19555	243
76143	The Netherlands	Europe	2019	13292	19641	16
76144	The Netherlands	Europe	2019	1588	19642	21
76145	The Netherlands	Europe	2019	1580	2493	16
76146	The Netherlands	Europe	2019	15575	5	18
76147	The Netherlands	Europe	2019	13292	9208	16
76148	The Netherlands	Europe	2019	7822	14994	3
76149	The Netherlands	Europe	2019	1583	15589	133
76150	The Netherlands	Europe	2019	1588	15956	21
76151	The Netherlands	Europe	2019	10314	19188	3
76152	The Netherlands	Europe	2019	7822	14994	3
76153	The Netherlands	Europe	2019	12973	19643	62
76154	The Netherlands	Europe	2019	9363	18146	16
76155	The Netherlands	Europe	2019	8163	8110	439
76156	The Netherlands	Europe	2019	9363	17495	16
76157	The Netherlands	Europe	2019	9903	18354	41
76158	The Netherlands	Europe	2019	11990	51	414
76159	The Netherlands	Europe	2019	7827	18666	219
76160	The Netherlands	Europe	2019	8156	5441	17
76161	The Netherlands	Europe	2019	7367	19644	16
76162	The Netherlands	Europe	2019	7827	16441	219
76163	The Netherlands	Europe	2019	7827	8845	219
76164	The Netherlands	Europe	2019	1599	11461	243
76165	The Netherlands	Europe	2019	1599	11461	243
76166	The Netherlands	Europe	2019	8143	19645	62
76167	The Netherlands	Europe	2019	8163	2	439
76168	The Netherlands	Europe	2019	7822	14994	3
76169	The Netherlands	Europe	2019	10314	19646	3
76170	The Netherlands	Europe	2019	10314	19188	3

76171	The Netherlands	Europe	2019	14253	16065	3
76172	The Netherlands	Europe	2019	7359	18526	223
76173	The Netherlands	Europe	2019	11864	18234	16
76174	The Netherlands	Europe	2019	1583	15589	133
76175	The Netherlands	Europe	2019	8143	19647	62
76176	The Netherlands	Europe	2019	7827	10386	219
76177	The Netherlands	Europe	2019	13334	12004	17
76178	The Netherlands	Europe	2019	13956	13893	17
76179	The Netherlands	Europe	2019	1583	15589	133
76180	The Netherlands	Europe	2019	11990	51	414
76181	The Netherlands	Europe	2019	9363	12302	16
76182	The Netherlands	Europe	2019	7827	19233	219
76183	The Netherlands	Europe	2019	11706	17972	3
76184	The Netherlands	Europe	2019	11422	19648	16
76185	The Netherlands	Europe	2019	7827	19649	219
76186	The Netherlands	Europe	2019	1588	13234	21
76187	The Netherlands	Europe	2019	8135	387	425
76188	The Netherlands	Europe	2019	7367	19644	16
76189	The Netherlands	Europe	2019	15331	19650	3
76193	The Netherlands	Europe	2017	7827	10386	219
76194	The Netherlands	Europe	2017	7827	10386	219
76195	The Netherlands	Europe	2017	7827	14051	219
76196	The Netherlands	Europe	2018	1901	1407	3
76197	The Netherlands	Europe	2018	1901	1407	3
76198	The Netherlands	Europe	2018	1901	1407	3
76199	The Netherlands	Europe	2018	7827	10386	219
76200	The Netherlands	Europe	2018	7827	2318	219
76201	The Netherlands	Europe	2018	7827	2318	219
76202	The Netherlands	Europe	2018	7827	2318	219
76203	The Netherlands	Europe	2018	7827	19651	219
76204	The Netherlands	Europe	2018	7827	14051	219
76205	The Netherlands	Europe	2018	7827	14051	219
76206	The Netherlands	Europe	2018	7827	10386	219
76207	The Netherlands	Europe	2018	7827	10386	219
76208	The Netherlands	Europe	2018	7827	10386	219
76209	The Netherlands	Europe	2018	7827	16441	219
76210	The Netherlands	Europe	2018	7827	10386	219
76211	The Netherlands	Europe	2018	7827	18113	219
76212	The Netherlands	Europe	2019	7827	19652	219
76213	The Netherlands	Europe	2019	7827	19652	219
76214	The Netherlands	Europe	2019	7827	16441	219
76215	The Netherlands	Europe	2019	7827	10386	219

76216	The Netherlands	Europe	2019	7827	16441	219
76217	The Netherlands	Europe	2019	7827	10386	219
76218	The Netherlands	Europe	2019	7827	16441	219
76219	The Netherlands	Europe	2019	7827	10386	219
76220	The Netherlands	Europe	2019	7827	10386	219
76221	The Netherlands	Europe	2019	7827	10386	219
76222	The Netherlands	Europe	2019	7827	10386	219
76223	The Netherlands	Europe	2019	7827	10386	219
76224	The Netherlands	Europe	2019	7827	16441	219
76225	The Netherlands	Europe	2019	7827	10386	219
76226	The Netherlands	Europe	2019	14369	14051	219
76227	The Netherlands	Europe	2019	14369	14051	219
76228	The Netherlands	Europe	2009	1901	1407	3
76229	The Netherlands	Europe	2010	1901	1407	3
76230	The Netherlands	Europe	2010	8114	4	44
76231	The Netherlands	Europe	2010	1901	1407	3
76232	The Netherlands	Europe	2010	1901	1407	3
76233	The Netherlands	Europe	2010	1901	1407	3
76234	The Netherlands	Europe	2010	1901	1407	3
76235	The Netherlands	Europe	2010	1901	1407	3
76236	The Netherlands	Europe	2010	1901	1407	3
76237	The Netherlands	Europe	2010	1901	1407	3
76238	The Netherlands	Europe	2011	1579	21	24
76239	The Netherlands	Europe	2012	7363	2400	196
76240	The Netherlands	Europe	2012	1901	1407	3
76241	The Netherlands	Europe	2012	7363	2400	196
76242	The Netherlands	Europe	2012	1901	13846	3
76243	The Netherlands	Europe	2012	1901	1407	3
76244	The Netherlands	Europe	2012	1901	3128	3
76245	The Netherlands	Europe	2012	7363	2400	196
76246	The Netherlands	Europe	2012	1901	1407	3
76247	The Netherlands	Europe	2013	1901	13846	3
76248	The Netherlands	Europe	2013	1901	1407	3
76249	The Netherlands	Europe	2013	1579	1034	24
76250	The Netherlands	Europe	2013	7363	2400	196
76251	The Netherlands	Europe	2013	7363	2400	196
76252	The Netherlands	Europe	2013	1579	21	24
76253	The Netherlands	Europe	2013	1579	21	24
76254	The Netherlands	Europe	2013	7363	2400	196
76255	The Netherlands	Europe	2013	7363	8435	196
76256	The Netherlands	Europe	2013	1901	1407	3
76257	The Netherlands	Europe	2013	7363	3811	196

76258	The Netherlands	Europe	2013	7363	14358	196
76259	The Netherlands	Europe	2013	7360	1407	3
76260	The Netherlands	Europe	2013	1901	6827	
76261	The Netherlands	Europe	2013	7363	10257	196
76262	The Netherlands	Europe	2013	7363	2400	196
76263	The Netherlands	Europe	2013	1901	3806	3
76264	The Netherlands	Europe	2013	7363	2400	196
76265	The Netherlands	Europe	2013	7363	10257	196
76266	The Netherlands	Europe	2013	7363	10257	196
76267	The Netherlands	Europe	2013	7363	10257	196
76268	The Netherlands	Europe	2014	1579	13407	24
76269	The Netherlands	Europe	2014	1579	11064	24
76270	The Netherlands	Europe	2014	7363	2400	196
76271	The Netherlands	Europe	2014	7363	2400	196
76272	The Netherlands	Europe	2014	7363	2400	196
76273	The Netherlands	Europe	2014	7363	6360	196
76274	The Netherlands	Europe	2014	7363	2400	196
76275	The Netherlands	Europe	2014	7363	6360	196
76276	The Netherlands	Europe	2014	1579	21	24
76277	The Netherlands	Europe	2014	7363	10257	196
76278	The Netherlands	Europe	2014	1579	21	24
76279	The Netherlands	Europe	2014	1901	1407	3
76280	The Netherlands	Europe	2015	7367	1407	3
76281	The Netherlands	Europe	2015	7363	11800	196
76282	The Netherlands	Europe	2015	7363	19653	196
76283	The Netherlands	Europe	2015	1579	21	24
76284	The Netherlands	Europe	2015	1901	2212	3
76286	The Netherlands	Europe	2010	7363	14343	196
76287	The Netherlands	Europe	2011	1901	3128	3
76288	The Netherlands	Europe	2011	1901	5533	3
76289	The Netherlands	Europe	2013	1901	3806	3
76290	The Netherlands	Europe	2013	7363	10257	196
76291	The Netherlands	Europe	2014		1034	
76292	The Netherlands	Europe	2014	7363	6360	196
76293	The Netherlands	Europe	2017	9363	12302	16
76323	Norway	Europe	2017	7363	19040	239
76333	Norway	Europe	2017	10622	15920	41
76348	Norway	Europe	2017	13415	12547	3
76407	Norway	Europe	2017	13484	5793	516
76455	Norway	Europe	2017	1600		533
76473	Norway	Europe	2017	13333		239
76497	Norway	Europe	2017	10932	12302	16

76511	Norway	Europe	2017	8776	19176	354
78483	Canada	North America	1997	1904	10476	432
78490	Canada	North America	2001	12524	108	370
78505	Canada	North America	2006	12531	1056	133
78507	Canada	North America	2006	1590	4014	435
78510	Canada	North America	2006	8153	3108	3
78514	Canada	North America	2006	1901	225	18
78519	Canada	North America	2007	12532	3565	31
78550	Canada	North America	2010	10891	757	3
78557	Canada	North America	2010	12537	51	414
78558	Canada	North America	2010	12536	6351	44
78578	Canada	North America	2010	11427	3150	3
78592	Canada	North America	2011	9365	1513	3
78594	Canada	North America	2011	10634	7159	532
78599	Canada	North America	2011	12542	1407	3
78611	Canada	North America	2012	1600	8851	151
78632	Canada	North America	2012	10931	3656	422
78637	Canada	North America	1989	1927	919	409
79079	USA	North America	2016	13539	17683	304
79114	USA	North America	2016	12093	13528	418
79117	USA	North America	2016	11422	4822	16
79124	USA	North America	2016	1600	4990	398
79137	USA	North America	2016	7367	17909	16
79140	USA	North America	2016	13537	4270	3
79155	USA	North America	2016	7371	3169	34
79174	USA	North America	2016	7822	14994	3
79210	USA	North America	2016	1904	7555	219
79219	USA	North America	2016	13548	7974	196
79223	USA	North America	2016	13413	15745	509
79224	USA	North America	2016	12086	25	414
79233	USA	North America	2016	11967	5004	304
79260	USA	North America	2016	6959	17721	304
79273	USA	North America	2016	13526	9918	62
79282	USA	North America	2016	1601	6339	509
79283	USA	North America	2016	13536	5441	17
79297	USA	North America	2016	13532	9296	509
79315	USA	North America	2016	13489	14700	219
79316	USA	North America	2016	11181	17730	418
79318	USA	North America	2016	12974	17731	219
79332	USA	North America	2016	13547	17708	16
79336	USA	North America	2016	8154	17712	44
79360	USA	North America	2016	12462	6968	244

79369	USA	North America	2016	11986	28	41
82283	USA	North America	2004	7371	292	3
82290	USA	North America	2011	10312	1407	3
83824	Norway	Europe	2018	13942	19055	37
83853	Norway	Europe	2018	14108	7574	196
86565	New Zealand	Oceania	2015	11428	9368	16
86566	New Zealand	Oceania	2015	13529	766	17
86634	New Zealand	Oceania	2015	11463	13990	16
86685	New Zealand	Oceania	2015	12040	7803	523
86840	New Zealand	Oceania	2015	14464	13990	16
86860	New Zealand	Oceania	2015	10932	7577	304
86914	New Zealand	Oceania	2014	14446		133

Table S2. List of accessory NEIS loci with its prevalence and functional annotation.

NEIS locus	Duplicate NEIS loci	Prevalence in subset of 765 isolates	Prevalence in all 8013 isolates	(PubMLST) gene annotation	PubMLST functional scheme (NG schemes only)	Final annotation	Identified by
NEIS0027	NEIS1865	3.7%	3.6%	hypothetical integral membrane protein	-	Nf1 phage island	Genome Comparator + PIRATE
NEIS0028	NEIS1864	3.7%	3.6%	hypothetical integral membrane protein	-	Nf1 phage island	Genome Comparator + PIRATE
NEIS0029	NEIS1863/ NEIS2453/ NEIS2459	3.7%	3.6%	hypothetical protein	-	Nf1 phage island	Genome Comparator + PIRATE
NEIS0030	NEIS1862/ NEIS2452/ NEIS2460	3.7%	3.6%	conserved hypothetical protein	-	Nf1 phage island	Genome Comparator + PIRATE
NEIS0031	NEIS1861/ NEIS0277	3.7%	3.6%	putative phage replication initiation factor	-	Nf1 phage island	Genome Comparator + PIRATE
NEIS0080		38.4%	48.7%	hypothetical protein	Other loci	Putative secretion system	Genome Comparator + PIRATE
NEIS0081		38.4%	48.7%	hypothetical protein	Other loci	Putative secretion system	Genome Compator
NEIS0083		37.8%	48.0%	hypothetical protein	Other loci	Putative secretion system	Genome Comparator + PIRATE
NEIS0084		37.9%	48.4%	hypothetical protein	Other loci	Putative secretion system	Genome Compator
NEIS0085		38.4%	48.6%	protein export protein	Other loci	Putative secretion system	Genome Comparator + PIRATE
NEIS0086		38.2%	48.4%	protein export protein	Other loci	Putative secretion system	Genome Comparator + PIRATE
NEIS0089		38.4%	48.7%	hypothetical protein	Other loci	Putative secretion system	Genome Comparator + PIRATE

NEIS0364		78.3%	79.6%	hypothetical protein	Other loci	Hypothetical protein	Genome Comparator + PIRATE
NEIS0365		78.3%	79.3%	hypothetical protein	Other loci	Hypothetical protein	Genome Comparator + PIRATE
NEIS0486		30.1%	26.0%	alcohol dehydrogenase	-	Alcohol dehydrogenase	Genome Comparator + PIRATE
NEIS0524		52.3%	74.4%	putative peptidase	-	Putative peptidase	Genome Compator
NEIS0594		38.2%	35.0%	hypothetical protein	Other loci	Hypothetical protein	Genome Comparator + PIRATE
NEIS0840		1.2%	0.8%	phage related protein	-	(Putative) phage associated protein	Genome Compator
NEIS0953	NEIS1664	27.6%	17.3%	hypothetical protein	-	(Putative) phage associated protein	Genome Comparator + PIRATE
NEIS0955		80.4%	92.0%	hypothetical protein	Other loci	Nf4 phage island	Genome Comparator + PIRATE
NEIS1089		28.5%	26.7%	hypothetical protein	-	Hypothetical protein	Genome Compator
NEIS1193		91.1%	92.1%	type III restriction-modification system endonuclease protein	Methyltransferase s	Methyltransferase	Genome Comparator + PIRATE
NEIS1194		90.8%	90.7%	Type III restriction/modification system methyltransferase	Methyltransferase s	Methyltransferase	Genome Comparator + PIRATE
NEIS1311		85.0%	84.0%	putative type III restriction/modification system enzyme	Methyltransferase s	Methyltransferase	Genome Compator
NEIS1667		22.9%	12.4%	hypothetical protein	-	Nf4 phage island	Genome Comparator + PIRATE
NEIS2202		36.9%	26.5%	DNA primase	Conjugative Plasmid	Conjugative Plasmid	Genome Comparator + PIRATE

NEIS2203	37.5%	27.1%	transcription elongation factor	Conjugative Plasmid	Conjugative Plasmid	Genome Comparator + PIRATE
NEIS2204	37.6%	27.2%	yegA / hypothetical protein	Conjugative Plasmid	Conjugative Plasmid	Genome Comparator + PIRATE
NEIS2205	37.6%	27.2%	hypothetical protein	Conjugative Plasmid	Conjugative Plasmid	Genome Comparator + PIRATE
NEIS2206	37.3%	27.1%	hypothetical protein	Conjugative Plasmid	Conjugative Plasmid	Genome Comparator + PIRATE
NEIS2207	37.5%	27.2%	hypothetical protein	Conjugative Plasmid	Conjugative Plasmid	Genome Comparator + PIRATE
NEIS2208	37.0%	26.9%	putative DNA modification methylase	Conjugative Plasmid/ Methyltransferases	Conjugative Plasmid	Genome Comparator + PIRATE
NEIS2209	37.4%	27.1%	hypothetical protein	Conjugative Plasmid	Conjugative Plasmid	Genome Comparator + PIRATE
NEIS2210	22.9%	14.3%	tetM / tetracyclin resistance protein	Conjugative Plasmid	Conjugative Plasmid	Genome Comparator + PIRATE
NEIS2211	22.6%	13.8%	hypothetical protein	Conjugative Plasmid	Conjugative Plasmid	Genome Comparator + PIRATE
NEIS2212	22.4%	13.8%	epsilon_2 antitoxin	Conjugative Plasmid	Conjugative Plasmid	Genome Comparator + PIRATE
NEIS2213	37.5%	27.0%	zeta_2 toxin	Conjugative Plasmid	Conjugative Plasmid	Genome Comparator + PIRATE
NEIS2214	37.8%	27.3%	-	Conjugative Plasmid	Conjugative Plasmid	Genome Comparator + PIRATE
NEIS2215	37.3%	26.9%	zeta_1 toxin	Conjugative Plasmid	Conjugative Plasmid	Genome Comparator + PIRATE
NEIS2216	37.6%	27.2%	marR / transcriptional regulator	Conjugative Plasmid	Conjugative Plasmid	Genome Comparator + PIRATE
NEIS2217	37.5%	27.1%	res / putative resolvase	Conjugative Plasmid	Conjugative Plasmid	Genome Comparator + PIRATE

NEIS2218	37.5%	27.2%	vapD / toxin	Conjugative Plasmid	Conjugative Plasmid	Genome Comparator + PIRATE
NEIS2219	37.5%	27.0%	trbN / lytic transglycosylase	Conjugative Plasmid	Conjugative Plasmid	Genome Comparator + PIRATE
NEIS2220	37.8%	27.0%	trbM / involved in conjugative transfer	Conjugative Plasmid	Conjugative Plasmid	Genome Comparator + PIRATE
NEIS2221	37.3%	26.9%	trbL / virB6-like inner membrane protein	Conjugative Plasmid	Conjugative Plasmid	Genome Compator
NEIS2222	37.5%	26.6%	trbK / putative entry exclusion protein	Conjugative Plasmid	Conjugative Plasmid	Genome Compator
NEIS2223	37.4%	27.1%	trbJ	Conjugative Plasmid	Conjugative Plasmid	Genome Comparator + PIRATE
NEIS2224	37.3%	26.8%	trbI / virbB10-like core complex component	Conjugative Plasmid	Conjugative Plasmid	Genome Compator
NEIS2225	37.6%	27.2%	trbH / virB7-like core complex component	Conjugative Plasmid	Conjugative Plasmid	Genome Comparator + PIRATE
NEIS2226	37.3%	26.9%	trbG / virB9-like core complex component	Conjugative Plasmid	Conjugative Plasmid	Genome Comparator + PIRATE
NEIS2227	37.5%	27.1%	trbF / DNA transfer protein	Conjugative Plasmid	Conjugative Plasmid	Genome Comparator + PIRATE
NEIS2228	36.9%	26.8%	trbE / virB4-like conjugal transfer ATPase	Conjugative Plasmid	Conjugative Plasmid	Genome Comparator + PIRATE
NEIS2229	37.8%	27.2%	trbD / similar to VirB3	Conjugative Plasmid	Conjugative Plasmid	Genome Comparator + PIRATE
NEIS2230	37.8%	27.2%	trbC / virB2 prepilin	Conjugative Plasmid	Conjugative Plasmid	Genome Comparator + PIRATE
NEIS2231	37.3%	26.8%	trbB / virB11-like conjugal transfer ATPase	Conjugative Plasmid	Conjugative Plasmid	Genome Comparator + PIRATE
NEIS2232	37.9%	27.2%	trbA / transcriptional repressor	Conjugative Plasmid	Conjugative Plasmid	Genome Comparator + PIRATE
NEIS2233	37.6%	27.2%	ssb / putative single stranded DNA binding protein	Conjugative Plasmid	Conjugative Plasmid	Genome Comparator + PIRATE

NEIS2234	37.4%	27.1%	trfA / oriV activator protein	Conjugative Plasmid	Conjugative Plasmid	Genome Comparator + PIRATE
NEIS2235	37.8%	27.2%	korC / regulator of plasmid partitioning	Conjugative Plasmid	Conjugative Plasmid	Genome Comparator + PIRATE
NEIS2236	37.6%	27.2%	kleE / involved in plasmid partitioning	Conjugative Plasmid	Conjugative Plasmid	Genome Comparator + PIRATE
NEIS2237	37.5%	27.1%	incC2 / ATP binding protein	Conjugative Plasmid	Conjugative Plasmid	Genome Comparator + PIRATE
NEIS2238	37.4%	27.1%	parB / DNA binding protein	Conjugative Plasmid	Conjugative Plasmid	Genome Comparator + PIRATE
NEIS2239	35.2%	26.5%	kfrB / putative kfrB/traO	Conjugative Plasmid	Conjugative Plasmid	Genome Comparator + PIRATE
NEIS2240	37.6%	27.2%	kfrC / putative kfrC/traN protein	Conjugative Plasmid	Conjugative Plasmid	Genome Comparator + PIRATE
NEIS2241	37.4%	27.0%	traM / involved in conjugal transfer	Conjugative Plasmid	Conjugative Plasmid	Genome Comparator + PIRATE
NEIS2242	37.4%	27.1%	traL / involved in conjugal transfer	Conjugative Plasmid	Conjugative Plasmid	Genome Comparator + PIRATE
NEIS2243	37.5%	27.2%	traK / involved in conjugal transfer	Conjugative Plasmid	Conjugative Plasmid	Genome Comparator + PIRATE
NEIS2244	37.9%	27.2%	traJ / oriT	Conjugative Plasmid	Conjugative Plasmid	Genome Comparator + PIRATE
NEIS2245	37.1%	26.6%	traI / DNA relaxase	Conjugative Plasmid	Conjugative Plasmid	Genome Comparator + PIRATE
NEIS2246	36.7%	26.4%	traG / conjugal coupling protein	Conjugative Plasmid	Conjugative Plasmid	Genome Comparator + PIRATE
NEIS2247	37.5%	27.2%	traF / conjugal prepilin peptidase	Conjugative Plasmid	Conjugative Plasmid	Genome Comparator + PIRATE
NEIS2248	37.5%	26.9%	traD / DNA topoisomerase III	Conjugative Plasmid	Conjugative Plasmid	Genome Comparator + PIRATE
NEIS2249	37.5%	27.1%	traD / involved in conjugal transfer	Conjugative Plasmid	Conjugative Plasmid	Genome Comparator + PIRATE

NEIS2250	63.9%	69.0%	traD / putative docking protein	Gonococcal Genetic Island	Gonococcal Genetic Island	Genome Comparator + PIRATE
NEIS2251	63.8%	68.9%	traI / putative nicking enzyme	Gonococcal Genetic Island	Gonococcal Genetic Island	Genome Comparator + PIRATE
NEIS2252	63.9%	68.9%	yaf / hypothetical protein	Gonococcal Genetic Island	Gonococcal Genetic Island	Genome Comparator + PIRATE
NEIS2253	63.9%	68.9%	ltgX / peptidoglycan hydrolase	Gonococcal Genetic Island	Gonococcal Genetic Island	Genome Comparator + PIRATE
NEIS2254	63.9%	69.0%	yag / outer membrane protein	Gonococcal Genetic Island	Gonococcal Genetic Island	Genome Comparator + PIRATE
NEIS2255	64.2%	69.0%	traA / putative transfer protein	Gonococcal Genetic Island	Gonococcal Genetic Island	Genome Comparator + PIRATE
NEIS2256	64.1%	69.0%	traL / pilus assembly	Gonococcal Genetic Island	Gonococcal Genetic Island	Genome Comparator + PIRATE
NEIS2257	63.9%	69.0%	traE / pilus biogenesis	Gonococcal Genetic Island	Gonococcal Genetic Island	Genome Comparator + PIRATE
NEIS2258	63.8%	68.9%	traK / pilus assembly	Gonococcal Genetic Island	Gonococcal Genetic Island	Genome Comparator + PIRATE
NEIS2259	63.8%	68.8%	traB / conjugal transfer	Gonococcal Genetic Island	Gonococcal Genetic Island	Genome Comparator + PIRATE
NEIS2260	63.9%	68.9%	dsbC / protein disulphide isomerase	Gonococcal Genetic Island	Gonococcal Genetic Island	Genome Comparator + PIRATE
NEIS2261	63.9%	68.9%	traV / putative transfer protein	Gonococcal Genetic Island	Gonococcal Genetic Island	Genome Comparator + PIRATE
NEIS2262	63.7%	68.7%	traC / pilus assembly	Gonococcal Genetic Island	Gonococcal Genetic Island	Genome Comparator + PIRATE
NEIS2263	63.9%	69.0%	ybe / hypothetical protein	Gonococcal Genetic Island	Gonococcal Genetic Island	Genome Comparator + PIRATE
NEIS2264	64.1%	69.0%	trbI / conjugal transfer	Gonococcal Genetic Island	Gonococcal Genetic Island	Genome Comparator + PIRATE
NEIS2265	63.9%	68.9%	traW / pilus biogenesis	Gonococcal Genetic Island	Gonococcal Genetic Island	Genome Comparator + PIRATE

NEIS2266	63.1%	68.8%	traU / pulus biogenesis	Gonococcal Genetic Island	Gonococcal Genetic Island	Genome Comparator + PIRATE
NEIS2267	63.7%	68.7%	trbC / conjugative transfer	Gonococcal Genetic Island	Gonococcal Genetic Island	Genome Comparator + PIRATE
NEIS2268	63.8%	68.8%	ybi / mating-pair stabilisation	Gonococcal Genetic Island	Gonococcal Genetic Island	Genome Comparator + PIRATE
NEIS2269	63.5%	68.7%	traN / mating-pair stabilisation	Gonococcal Genetic Island	Gonococcal Genetic Island	Genome Comparator + PIRATE
NEIS2270	63.8%	68.8%	ycb / hypothetical protein	Gonococcal Genetic Island	Gonococcal Genetic Island	Genome Comparator + PIRATE
NEIS2271	63.5%	68.8%	traF / pilus assembly	Gonococcal Genetic Island	Gonococcal Genetic Island	Genome Comparator + PIRATE
NEIS2272	63.8%	68.8%	traH / pilus assembly	Gonococcal Genetic Island	Gonococcal Genetic Island	Genome Comparator + PIRATE
NEIS2273	63.4%	68.8%	traG / pilus assembly; mating-pair stabilisation	Gonococcal Genetic Island	Gonococcal Genetic Island	Genome Comparator + PIRATE
NEIS2274	58.0%	61.5%	atlA / peptidoglycan transglycosylase	Gonococcal Genetic Island	Gonococcal Genetic Island	Genome Comparator + PIRATE
NEIS2275	58.2%	61.6%	ych / hypothetical protein	Gonococcal Genetic Island	Gonococcal Genetic Island	Genome Comparator + PIRATE
NEIS2276	57.9%	61.1%	exp1 / exported protein	Gonococcal Genetic Island	Gonococcal Genetic Island	Genome Compator
NEIS2277	59.5%	62.1%	cspA / RNA/ssDNA binding protein	Gonococcal Genetic Island	Gonococcal Genetic Island	Genome Comparator + PIRATE
NEIS2278	57.3%	60.1%	exp2 / hypothetical protein	Gonococcal Genetic Island	Gonococcal Genetic Island	Genome Comparator + PIRATE
NEIS2279	63.5%	68.3%	yda / hypothetical protein	Gonococcal Genetic Island	Gonococcal Genetic Island	Genome Comparator + PIRATE
NEIS2280	63.3%	68.9%	ydbA / hypothetical protein	Gonococcal Genetic Island	Gonococcal Genetic Island	Genome Comparator + PIRATE
NEIS2281	63.7%	68.9%	ydbB / hypothetical protein	Gonococcal Genetic Island	Gonococcal Genetic Island	Genome Comparator + PIRATE

NEIS2282	63.9%	69.1%	ydcA / putative protease	Gonococcal Genetic Island	Gonococcal Genetic Island	Genome Comparator + PIRATE
NEIS2283	63.8%	69.1%	ydcB / hypothetical protein	Gonococcal Genetic Island	Gonococcal Genetic Island	Genome Comparator + PIRATE
NEIS2284	63.9%	69.1%	ydd / hypothetical protein	Gonococcal Genetic Island	Gonococcal Genetic Island	Genome Comparator + PIRATE
NEIS2285	63.9%	69.2%	ydeA / hypothetical protein	Gonococcal Genetic Island	Gonococcal Genetic Island	Genome Comparator + PIRATE
NEIS2286	63.8%	69.0%	ydeB / hypothetical protein	Gonococcal Genetic Island	Gonococcal Genetic Island	Genome Comparator + PIRATE
NEIS2287	63.7%	69.0%	ydf / hypothetical protein	Gonococcal Genetic Island	Gonococcal Genetic Island	Genome Comparator + PIRATE
NEIS2288	63.9%	69.0%	ydg / putative DNA methylase	Gonococcal Genetic Island/ Methyltransferase s	Gonococcal Genetic Island	Genome Comparator + PIRATE
NEIS2289	63.1%	68.9%	ydhA / putative DNA methylase	Gonococcal Genetic Island/ Methyltransferase s	Gonococcal Genetic Island	Genome Comparator + PIRATE
NEIS2290	63.8%	69.1%	ydhB / hypothetical protein	Gonococcal Genetic Island	Gonococcal Genetic Island	Genome Comparator + PIRATE
NEIS2291	63.7%	69.0%	ydi / hypothetical protein	Gonococcal Genetic Island	Gonococcal Genetic Island	Genome Comparator + PIRATE
NEIS2292	62.9%	68.7%	yea / putative helicase	Gonococcal Genetic Island	Gonococcal Genetic Island	Genome Comparator + PIRATE
NEIS2293	61.6%	63.1%	yeb / putative N-acetyltransferase	Gonococcal Genetic Island	Gonococcal Genetic Island	Genome Comparator + PIRATE
NEIS2294	63.7%	69.0%	yecA / hypothetical protein	Gonococcal Genetic Island	Gonococcal Genetic Island	Genome Comparator + PIRATE
NEIS2295	62.0%	67.2%	yecB / repeat containing protein	Gonococcal Genetic Island	Gonococcal Genetic Island	Genome Comparator + PIRATE

NEIS2296	63.8%	69.1%	yedA / hypothetical protein	Gonococcal Genetic Island	Gonococcal Genetic Island	Genome Comparator + PIRATE
NEIS2297	63.9%	69.0%	yedB / hypothetical protein	Gonococcal Genetic Island	Gonococcal Genetic Island	Genome Comparator + PIRATE
NEIS2298	64.1%	69.1%	yee / hypothetical protein	Gonococcal Genetic Island	Gonococcal Genetic Island	Genome Compator
NEIS2299	63.8%	69.1%	yegA / hypothetical protein	Gonococcal Genetic Island	Gonococcal Genetic Island	Genome Comparator + PIRATE
NEIS2300	63.9%	69.2%	yegB / hypothetical protein	Gonococcal Genetic Island	Gonococcal Genetic Island	Genome Compator
NEIS2301	61.2%	67.8%	yeh / hypothetical protein	Gonococcal Genetic Island	Gonococcal Genetic Island	Genome Comparator + PIRATE
NEIS2302	63.7%	68.9%	topB / DNA topoisomerase	Gonococcal Genetic Island	Gonococcal Genetic Island	Genome Comparator + PIRATE
NEIS2303	64.2%	69.1%	ssbB / single-stranded DNA binding protein	Gonococcal Genetic Island	Gonococcal Genetic Island	Genome Comparator + PIRATE
NEIS2304	63.8%	68.6%	yfa / hypothetical protein	Gonococcal Genetic Island	Gonococcal Genetic Island	Genome Comparator + PIRATE
NEIS2305	63.9%	68.9%	yfb / hypothetical protein	Gonococcal Genetic Island	Gonococcal Genetic Island	Genome Comparator + PIRATE
NEIS2306	63.9%	68.9%	yfd / putative tonB-like transporter	Gonococcal Genetic Island	Gonococcal Genetic Island	Genome Comparator + PIRATE
NEIS2307	63.3%	69.0%	yfeA / hypothetical protein	Gonococcal Genetic Island	Gonococcal Genetic Island	Genome Comparator + PIRATE
NEIS2308	64.1%	68.9%	yfeB / hypothetical protein	Gonococcal Genetic Island	Gonococcal Genetic Island	Genome Comparator + PIRATE
NEIS2309	64.1%	69.0%	parB / chromosome partitioning	Gonococcal Genetic Island	Gonococcal Genetic Island	Genome Comparator + PIRATE
NEIS2310	63.5%	68.9%	parA / chromosome partitioning	Gonococcal Genetic Island	Gonococcal Genetic Island	Genome Comparator + PIRATE
NEIS2311	5.6%	7.5%	eppA / hypothetical protein	Gonococcal Genetic Island	Gonococcal Genetic Island	Genome Comparator + PIRATE

NEIS2312	5.6%	7.5%	ych1 / hypothetical protein	Gonococcal Genetic Island	Gonococcal Genetic Island	Genome Comparator + PIRATE
NEIS2313	64.1%	69.1%	hypothetical protein	Gonococcal Genetic Island	Gonococcal Genetic Island	Genome Comparator + PIRATE
NEIS2314	63.9%	69.1%	hypothetical protein	Gonococcal Genetic Island	Gonococcal Genetic Island	Genome Comparator + PIRATE
NEIS2315	64.1%	69.1%	conjugal transfer protein	Gonococcal Genetic Island	Gonococcal Genetic Island	Genome Comparator + PIRATE
NEIS2323	1.2%	0.7%	-	VirB T4SS	VirB T4SS	Genome Comparator + PIRATE
NEIS2324	1.2%	0.7%	-	VirB T4SS	VirB T4SS	Genome Comparator + PIRATE
NEIS2325	1.2%	0.6%	-	VirB T4SS	VirB T4SS	Genome Compator
NEIS2326	1.2%	0.6%	-	VirB T4SS	VirB T4SS	Genome Comparator + PIRATE
NEIS2327	1.2%	0.7%	-	VirB T4SS	VirB T4SS	Genome Comparator + PIRATE
NEIS2328	1.2%	0.7%	-	VirB T4SS	VirB T4SS	Genome Comparator + PIRATE
NEIS2329	1.2%	0.7%	-	VirB T4SS	VirB T4SS	Genome Comparator + PIRATE
NEIS2330	1.2%	0.7%	-	VirB T4SS	VirB T4SS	Genome Comparator + PIRATE
NEIS2331	1.2%	0.7%	-	VirB T4SS	VirB T4SS	Genome Comparator + PIRATE
NEIS2332	1.2%	0.7%	-	VirB T4SS	VirB T4SS	Genome Comparator + PIRATE
NEIS2333	1.2%	0.7%	-	VirB T4SS	VirB T4SS	Genome Comparator + PIRATE
NEIS2334	1.2%	0.7%	-	VirB T4SS	VirB T4SS	Genome Comparator + PIRATE
NEIS2335	1.2%	0.7%	-	VirB T4SS	VirB T4SS	Genome Comparator + PIRATE

NEIS2336	1.2%	0.7%	-	VirB T4SS	VirB T4SS	Genome Comparator + PIRATE
NEIS2337	1.2%	0.7%	-	VirB T4SS	VirB T4SS	Genome Comparator + PIRATE
NEIS2338	1.2%	0.7%	-	VirB T4SS	VirB T4SS	Genome Comparator + PIRATE
NEIS2339	1.2%	0.7%	-	VirB T4SS	VirB T4SS	Genome Comparator + PIRATE
NEIS2342	1.2%	0.7%	-	VirB T4SS	VirB T4SS	Genome Comparator + PIRATE
NEIS2343	1.2%	0.7%	-	VirB T4SS	VirB T4SS	Genome Comparator + PIRATE
NEIS2345	88.4%	93.5%	-	VirB T4SS	VirB T4SS	Genome Comparator + PIRATE
NEIS2348	1.2%	0.7%	-	VirB T4SS	VirB T4SS	Genome Comparator + PIRATE
NEIS2350	1.2%	0.7%	-	VirB T4SS	VirB T4SS	Genome Comparator + PIRATE
NEIS2351	1.2%	0.7%	-	VirB T4SS	VirB T4SS	Genome Comparator + PIRATE
NEIS2352	1.2%	0.7%	-	VirB T4SS	VirB T4SS	Genome Comparator + PIRATE
NEIS2353	1.2%	0.7%	-	VirB T4SS	VirB T4SS	Genome Compator
NEIS2354	1.2%	0.7%	-	VirB T4SS	VirB T4SS	Genome Comparator + PIRATE
NEIS2356	15.4%	13.5%	-	Conjugative Plasmid	Conjugative Plasmid	Genome Compator
NEIS2357	16.3%	10.6%	beta lactamase	Beta-lactamase plasmid	Beta-lactamase plasmid	Genome Comparator + PIRATE
NEIS2358	19.1%	11.1%	replicase A	Beta-lactamase plasmid	Beta-lactamase plasmid	Genome Comparator + PIRATE
NEIS2359	19.2%	11.3%	resolvase	Beta-lactamase plasmid	Beta-lactamase plasmid	Genome Comparator + PIRATE

NEIS2360		7.2%	3.4%	-	Beta-lactamase plasmid	Beta-lactamase plasmid	Genome Comparator + PIRATE
NEIS2451	NEIS2461	3.7%	3.6%	hypothetical protein	Other loci	Nf1 phage island	Genome Compator
NEIS2486		89.2%	90.8%	conserved hypothetical protein	Other loci	Hypothetical protein	Genome Comparator + PIRATE
NEIS2582		20.4%	19.2%	hypothetical protein	Other loci	Hypothetical protein	Genome Comparator + PIRATE
NEIS2615		49.0%	60.1%	hypothetical protein, putative phage associated protein	Other loci	(Putative) phage associated protein	Genome Comparator + PIRATE
NEIS2616		65.8%	80.5%	replicative DNA helicase	Other loci	(Putative) phage associated protein	Genome Comparator + PIRATE
NEIS2618		39.3%	64.6%	hypothetical protein, putative phage associated protein	Other loci	(Putative) phage associated protein	Genome Comparator + PIRATE
NEIS2619		50.5%	58.5%	hypothetical protein, putative phage associated protein	Other loci	(Putative) phage associated protein	Genome Comparator + PIRATE
NEIS2621		4.7%	5.9%	hypothetical protein, putative phage associated protein	Other loci	(Putative) phage associated protein	Genome Comparator + PIRATE
NEIS2640		26.0%	22.6%	hypothetical protein, putative phage associated protein	Other loci	(Putative) phage associated protein	Genome Compator
NEIS2643		49.8%	73.4%	hypothetical protein, putative phage associated protein	Other loci	(Putative) phage associated protein	Genome Comparator + PIRATE
NEIS2646		4.7%	2.7%	putative TonB-dependent receptor	Other loci	TonB dependent receptor	Genome Compator
NEIS2647		5.4%	3.8%	hypothetical protein	Other loci	TonB dependent receptor	Genome Compator

NEIS2666	82.6%	86.7%	hypothetical protein	Other loci	Hypothetical protein	Genome Comparator + PIRATE
NEIS2691	87.7%	88.5%	restriction endonuclease R.NgoMIII	Methyltransferases	Methyltransferase	Genome Comparator + PIRATE
NEIS2692	87.5%	88.5%	DNA cytosine methyltransferase M.NgoMIII	Methyltransferases	Methyltransferase	Genome Comparator + PIRATE
NEIS2693	82.5%	82.7%	conserved hypothetical protein, putative phage associated protein	Other loci	Nf4 phage island	Genome Comparator + PIRATE
NEIS2705	56.9%	50.0%	hypothetical protein	Other loci	Hypothetical protein	Genome Compator
NEIS2713	28.4%	22.3%	phage associated protein	Other loci	(Putative) phage associated protein	Genome Comparator + PIRATE
NEIS2714	55.2%	59.6%	phage associated protein	Other loci	(Putative) phage associated protein	Genome Comparator + PIRATE
NEIS2718	47.7%	68.3%	phage associated protein	Other loci	(Putative) phage associated protein	Genome Compator
NEIS2744	94.2%	94.2%	TonB dependent receptor (SLAM)	Other loci	TonB dependent receptor	Genome Compator
NEIS2859	8.8%	24.3%	hypothetical protein	Other loci	Hypothetical protein	Genome Comparator + PIRATE
NEIS2960	19.5%	12.1%	hypothetical protein	Beta-lactamase plasmid	Beta-lactamase plasmid	Genome Compator
NEIS2961	14.0%	8.3%	DNA strand transferase	Beta-lactamase plasmid	Beta-lactamase plasmid	Genome Comparator + PIRATE
NEIS2962	13.3%	8.8%	hypothetical protein	Beta-lactamase plasmid	Beta-lactamase plasmid	Genome Comparator + PIRATE
NEIS2964	19.0%	11.3%	hypothetical protein	Beta-lactamase plasmid	Beta-lactamase plasmid	Genome Comparator + PIRATE

NEIS3177	0.7%	1.5%	(Putative) phage associated protein	Phage island X	PIRATE
NEIS3178	26.7%	38.6%	Hypothetical protein	Hypothetical protein	PIRATE
NEIS3179	74.6%	93.6%	Hypothetical protein	Hypothetical protein	PIRATE
NEIS3180	85.9%	79.5%	Hypothetical protein	Hypothetical protein	PIRATE
NEIS3181	93.1%	89.5%	(Putative) phage associated protein	(Putative) phage associated protein	PIRATE
NEIS3182	40.0%	57.6%	Restriction modification enzyme	Methyltransferase	PIRATE
NEIS3183	78.3%	93.5%	Hypothetical protein	Hypothetical protein	PIRATE
NEIS3184	74.2%	70.8%	Histone deacetylase	DNA transcription	PIRATE
NEIS3186	0.5%	1.2%	(Putative) phage associated protein	Phage island X	PIRATE
NEIS3188	27.6%	32.9%	Type IV toxin-antitoxin system, putative AbiEii toxin	Toxin-antitoxin system	PIRATE
NEIS3189	87.1%	93.9%	TspB virulence factor	TspB virulence factor	PIRATE
NEIS3190	4.7%	2.3%	Hypothetical protein	Conjugative Plasmid	PIRATE
NEIS3192	17.8%	16.2%	Hypothetical protein	Gonococcal Genetic Island	PIRATE
NEIS3193	70.8%	80.8%	Hypothetical protein	Hypothetical protein	PIRATE
NEIS3195	0.7%	1.5%	Hypothetical protein	Phage island X	PIRATE
NEIS3196	89.8%	90.6%	Hypothetical protein	Hypothetical protein	PIRATE

NEIS3197	94.5%	94.4%	Hypothetical protein	Hypothetical protein	PIRATE
NEIS3198	59.7%	81.7%	Hypothetical protein	Hypothetical protein	PIRATE
NEIS3200	37.6%	30.1%	Hypothetical protein	Hypothetical protein	PIRATE
NEIS3202	1.6%	1.7%	DNA replication protein	DNA replication	PIRATE
NEIS3203	1.4%	2.0%	(Putative) phage associated protein	(Putative) phage associated protein	PIRATE
NEIS3204	0.7%	1.5%	(Putative) phage associated protein	Phage island X	PIRATE
NEIS3205	1.6%	2.1%	(Putative) phage associated protein	(Putative) phage associated protein	PIRATE
NEIS3206	44.3%	27.4%	Hypothetical protein	Hypothetical protein	PIRATE
NEIS3207	0.7%	1.6%	Hypothetical protein	Phage island X	PIRATE
NEIS3208	67.1%	71.0%	Hypothetical protein	Hypothetical protein	PIRATE
NEIS3209	61.0%	61.8%	Hypothetical protein	Hypothetical protein	PIRATE
NEIS3210	23.0%	23.8%	Hypothetical protein	Hypothetical protein	PIRATE
NEIS3211	26.3%	30.9%	Helix-turn-helix transcriptional regulator	DNA transcription	PIRATE
NEIS3212	1.2%	0.7%	Hypothetical protein	VirB T4SS	PIRATE
NEIS3213	2.4%	1.4%	DUF domain-containing protein	Hypothetical protein	PIRATE
NEIS3214	24.1%	20.3%	(Putative) phage associated protein	(Putative) phage associated protein	PIRATE

NEIS3215	84.8%	88.0%	Hypothetical protein	Hypothetical protein	PIRATE
NEIS3216	12.5%	13.8%	Hypothetical protein	Hypothetical protein	PIRATE
NEIS3217	1.8%	2.0%	DUF domain-containing protein	Hypothetical protein	PIRATE
NEIS3218	94.4%	94.4%	(Putative) phage associated protein	(Putative) phage associated protein	PIRATE
NEIS3219	1.4%	2.1%	Hypothetical protein	Hypothetical protein	PIRATE
NEIS3220	94.0%	89.7%	Helix-turn-helix transcriptional regulator	DNA transcription	PIRATE
NEIS3221	1.6%	2.2%	(Putative) phage associated protein	(Putative) phage associated protein	PIRATE
NEIS3222	0.7%	1.5%	Hypothetical protein	Phage island X	PIRATE
NEIS3223	24.1%	20.3%	Hypothetical protein	Hypothetical protein	PIRATE
NEIS3224	0.7%	1.6%	Hypothetical protein	Phage island X	PIRATE
NEIS3225	1.2%	0.7%	ssDNA binding protein	VirB T4SS	PIRATE
NEIS3226	0.8%	0.8%	Membrane protein	Membrane protein	PIRATE
NEIS3227	9.4%	7.3%	Helix-turn-helix transcriptional regulator	Conjugative Plasmid	PIRATE
NEIS3228	86.8%	94.0%	Hypothetical protein	Hypothetical protein	PIRATE
NEIS3230	1.2%	0.7%	Hypothetical protein	VirB T4SS	PIRATE
NEIS3231	1.2%	0.7%	Type II toxin-antitoxin system - Plasmid stabilization system protein - parE toxin	VirB T4SS	PIRATE
NEIS3232	27.7%	32.9%	Type II toxin-antitoxin system - vapC family protein	Toxin-antitoxin system	PIRATE

NEIS3233	94.6%	94.5%	Hypothetical protein	Hypothetical protein	PIRATE
NEIS3234	59.1%	93.8%	Hypothetical protein	Hypothetical protein	PIRATE

**Legend**	
	Phage associated
	Hypotethical proteins
	Mobile Genetic Elements
	Toxin-antitoxin system
	Methyltransferases
	Putative secretion system
	TonB dependent receptor
	DNA transcription
	Other functions

Table S3. Significance of associations between cgMLST and accessory elements.

		Gono-coccal Genetic Island			Con-jugative plasmid			Beta-lacta-mase plasmid			VirB T4SS			Nf1 phage island			Putative secr-etion system		
**CgMLST group**	**Num-ber of isolates**	**Prevalence**	**adj. p-value**	**sign.**	**Prevalence**	**adj. p-value**	**sign.**	**Prevalence**	**adj. p-value**	**sign.**	**Prevalence**	**adj. p-value**	**sign.**	**Prevalence**	**adj. p-value**	**sign.**	**Prevalence**	**adj. p-value**	**sign.**
**3**	1452	99%	1.89E-236	****	0%	1.89E-212	****	1%	9.56E-54	****	0%	0.0103	*	0%	7.33E-24	****	97%	0	****
**16**	1433	20%	0	****	0%	1.43E-216	****	0%	1.54E-74	****	0%	0.0105	*	0%	2.14E-23	****	92%	0	****
**18**	357	90%	6.93E-20	****	1%	9.91E-40	****	2%	1.42E-09	****	0%	1		15%	1.93E-18	****	0%	3.79E-104	****
**196**	311	100%	2.04E-47	****	4%	1.54E-24	****	0%	1.01E-12	****	0%	1		0%	0.0039	**	0%	5.57E-90	****
**21**	289	41%	1.12E-20	****	93%	8.68E-130	****	77%	3.11E-158	****	0%	1		0%	0.0087	**	0%	1.87E-83	****
**8**	267	52%	5.57E-07	****	0%	1.74E-33	****	0%	7.66E-12	****	0%	1		0%	0.0197	*	95%	2.79E-62	****
**133**	239	59%	0.4340		100%	4.49E-138	****	31%	5.35E-15	****	0%	1		0%	0.0693		93%	8.03E-49	****
**41**	234	99%	8.89E-33	****	100%	3.29E-132	****	48%	1.28E-44	****	0%	1		0%	0.0665		0%	1.71E-64	****
**223**	231	99%	2.47E-32	****	1%	6.78E-27	****	0%	1.73E-08	****	0%	1		0%	0.1080		0%	7.73E-66	****
**24**	204	100%	1.85E-31	****	2%	3.71E-19	****	0%	1.07E-08	****	0%	1		0%	0.2260		0%	6.1E-58	****
**414**	190	100%	3.49E-29	****	7%	2.79E-10	****	1%	1.61E-06	****	0%	1		0%	0		0%	9.19E-54	****
**17**	166	81%	0.1680		1%	6.35E-18	****	1%	0.0003	***	0%	1		0%	1		95%	1.05E-36	****
**219**	159	9%	9.39E-56	****	1%	1.64E-18	****	4%	1		0%	1		0%	1		0%	1.79E-44	****
**446**	159	100%	3.54E-24	****	100%	2.71E-90	****	1%	0	***	0%	1		0%	1		1%	2.32E-42	****
**31**	119	54%	0.1550		12%	0.0162	*	1%	5.65E-03	**	0%	1		0%	1		74%	3.71E-06	****
**62**	115	12%	7.73E-35	****	100%	1.87E-64	****	47%	4.59E-20	****	0%	1		0%	1		1%	1.32E-29	****
**516**	104	100%	4.37E-15	****	0%	1.35E-12	****	34%	1.44E-07	****	0%	1		0%	1		0%	2.1E-28	****
**243**	90	100%	7.91E-13	****	76%	2.35E-19	****	2%	1		0%	1		0%	1		0%	2.89E-24	****
**425**	74	100%	4.14E-10	****	49%	0.0251	*	3%	1		0%	1		1%	1		1%	1.01E-17	****
**415**	73	100%	7.61E-10	****	1%	1.22E-06	****	0%	0		0%	1		100%	1.02E-107	****	0%	3.36E-19	****
**450**	70	97%	1.14E-06	****	100%	2.43E-38	****	1%	1		1%	1		0%	1		0%	2.69E-18	****
**416**	63	8%	6.6E-22	****	100%	2.64E-34	****	33%	0.0005	***	0%	1		0%	1		0%	1.68E-16	****
**37**	52	100%	1.67E-06	****	88%	5.1E-18	****	79%	1.27E-27	****	0%	1		0%	1		2%	1.33E-11	****
**44**	50	100%	2.69E-06	****	14%	1		0%	1		0%	1		0%	1		0%	1.01E-12	****
**95**	47	32%	5.22E-05	****	66%	7.2E-06	****	4%	1		0%	1		0%	1		98%	2.38E-11	****
**34**	46	98%	0.0003	***	0%	2.01E-04	***	0%	1		0%	1		0%	1		89%	1.7E-06	****
**498**	42	100%	0.0001	****	100%	3.01E-22	****	17%	1		0%	1		0%	1		0%	2.13E-10	****
**439**	39	5%	1.24E-14	****	90%	7.48E-14	****	0%	1		0%	1		0%	1		0%	1.59E-09	****

**304**	38	97%	0.0059	**	100%	5.87E-20	****	3%	1		5%	1		89%	2.3E-43	****	0%	3.11E-09	****
**239**	35	9%	2.79E-11	****	100%	3.04E-18	****	83%	2.66E-20	****	0%	1		0%	1		91%	3.06E-05	****
**Other**	675																		
**No cgMLST assigne**	601																		
**Overall prevale**	**8013**	**69%**			**27%**			**11%**			**0.7%**			**3.6%**			**49%**		

Adjusted p-values were calculated by multiplying the original p-value by the number of core genome groups tested (n=250). *p<0.05; **p<0.01; ***p<0.001; ****p<0.0001

significantly less prevalent in cgMLST group compared to distribution among all isolates

significantly more prevalent in cgMLST group compared to distribution among all isolates

Table S4. Significance of associations between accessory elements and antimicrobial resistance against penicillin, tetracycline, ciprofloxacin, ceftriaxone, cefixime and azithromycin.

			Gonococcal genetic island			Conjugative plasmid			Betalactamase plasmid			virB T4SS			Nf1 phage island			Putative secretion system		
	MIC	Number of isolates	prevalence	adj. p-value	sign.	prevalence	adj. p-value	sign.	prevalence	adj. p-value	sign.	prevalence	adj. p-value	sign.	prevalence	adj. p-value	sign.	prevalence	adj. p-value	sign.
PEN S	≤0.06	117	88.89%	6.66E-13	****	31.63%	4.69E-07	****	0.02%	5.87E-132	****	6.84%	2.00E-06	****	6.83%	0.4890		16.24%	8.28E-14	****
PEN I	>0.06-1.0	1896	63.98%			23.42%			1.85%			1.21%			3.38%			51.85%		
PEN R	>1.0	1074	74.02%			32.96%			32.68%			0.19%			2.79%			53.26%		
TET S	≤0.5	774	74.29%	0.0002	***	23.00%	0.0474	*	8.14%	5.26E-05	****	2.45%	0.0003	***	3.62%	1		36.56%	1.12E-21	****
TET R	>0.5	2143	66.17%			27.95%			14.19%			0.56%			3.13%			56.98%		
CIP S	≤0.03	2098	65.3%	0.0034	**	26.02%	0.2568		5.82%	8.28E-26	****	1.76%	5.66E-12	****	3.43%	0.0030	**	41.56%	1.07E-21	****
CIP R	>0.06	2316	70.16%			28.76%			15.67%			0.00%			1.77%			56.26%		
CRO S	≤0.125	4868	67.46%	0.1224		25.84%	1		10.56%	0.9600		0.64%	1		2.28%	1		50.92%	0.1032	
CRO R	>0.125	22	90.9%			13.64%			0.00%			0.00%			0.00%			77.27%		
CFX S	≤0.125	4004	65.86%	2.08E-19	****	29.00%	5.50E-52	****	12.01%	7.86E-21	****	0.85%	0.2760		2.7%	3.37E-05	****	46.55%	6.84E-98	****
CFX R	>0.125	490	85.31%			2.04%			0.61%			0.00%			0.00%			93.06%		
AZI S	<1.0	3547	70.99%	1.78E-09	****	29.01%	2.89E-47	****	12.52%	2.19E-23	****	0.93%	0.1950		2.68%	0.2292		44.8%	7.08E-61	****
AZI R	≥1.0	898	59.91%			7.57%			2.34%			0.22%			1.45%			75.17%		

Adjusted p-values were calculated by multiplying the original p-value by the number of accessory elements tested (n=6).

PEN=penicillin; TET=tetracycline; CIP=ciprofloxacin; CRO=ceftriaxone; CFX=cefixime; AZI=azithromycin; S=susceptible; I= intermediate; R=resistant

*p<0.05; **p<0.01; ***p<0.001; ****p<0.0001

significantly more prevalent among susceptible isolates

significantly more prevalent among resistant isolates

Table S5. Significance of associations between accessory genes and AMR.

Locus	NEIS0364			NEIS0365			NEIS0486			NEIS0524			NEIS0594			NEIS0840		
Funtional annotation	Hypothetical protein			Hypothetical protein			Alcohol dehydrogenase			Putative peptidase			Hypothetical protein			(Putative) phage associat		
	prevalence	adj.p-value	sign.	prevalence	adj.p-value	sign.	prevalence	adj.p-value	sign.	prevalence	adj.p-value	sign.	prevalence	adj.p-value	sign.	prevalence	adj.p-value	sign.
**PEN S**	31.62%	3.42E-45	****	30.77%	3.74E-47	****	47.01%	8.42E-09	****	47.86%	1.76E-08	****	8.55%	6.83E-72	****	0.85%	0.9594	
**PEN I**	79.27%			79.01%			21.52%			77.11%			43.25%			1.64%		
**PEN R**	90.50%			90.69%			28.40%			74.49%			13.59%			0.47%		
**TET S**	59.82%	2.15E-69	****	59.95%	1.72E-68	****	39.15%	6.20E-23	****	68.09%	1.26E-07	****	40.31%	2.56E-06	****	1.55%	1	
**TET R**	90.15%			90.11%			19.88%			79.14%			29.35%			0.98%		
**CIP S**	63.11%	1.18E-173	****	62.77%	5.23E-165	****	29.74%	2.64E-11	****	73.69%	1		44.57%	2.57E-49	****	2.19%	1.44E-08	****
**CIP R**	95.60%			94.91%			20.25%			75.60%			23.23%			0.22%		
**CRO S**	81.72%	1		81.12%	1		22.62%	1		74.53%	1		35.09%	1		1.13%	1	
**CRO R**	95.45%			90.91%			22.73%			86.36%			22.73%			0.00%		
**CFX S**	79.57%	2.43E-28	****	79.17%	4.63E-21	****	26.30%	7.13E-41	****	76.72%	4.87E-16	****	34.37%	0.0037	**	1.17%	1	
**CFX R**	97.55%			95.51%			2.65%			92.24%			25.31%			0.20%		
**AZI S**	79.14%	1.17E-37	****	78.74%	3.99E-39	****	26.87%	7.76E-44	****	74.15%	4.06E-16	****	30.65%	9.28E-19	****	1.04%	1	
**AZI R**	95.77%			95.77%			6.68%			87.19%			47.44%			1.00%		

Adjusted p-values were calculated by multiplying the original p-value by the number of genes tested (n=78)

*p<0.05; **p<0.01; ***p<0.001; ****p<0.0001

(blue) significantly more prevalent among susceptible isolates

(orange) significantly more prevalent among resistant isolates

Locus	NEIS0953			NEIS0955			NEIS1089			NEIS1193			NEIS1194			NEIS1311		
Funtional annotation	(Putative) phage associate			Nf4 phage island			Hypothetical protein			Methyltransferase			Methyltransferase			Methyltransferase		
	prevalence	adj.p-value	sign.	prevalence	adj.p-value	sign.	prevalence	adj.p-value	sign.	prevalence	adj.p-value	sign.	prevalence	adj.p-value	sign.	prevalence	adj.p-value	sign.
PEN S	15.38%	1.05E-05	****	82.91%	0.00081	***	22.22%	0.44538	**	83.76%	2.85E-06	****	83.76%	5.02E-07	****	54.70%	8.74E-52	****
PEN I	19.67%			94.30%			22.05%			90.77%			88.92%			79.80%		
PEN R	11.82%			95.44%			27.28%			95.53%			94.79%			96.28%		
TET S	21.71%	1.05E-03	**	90.70%	7.75E-05	****	25.06%	1		91.73%	1		90.96%	1		56.20%	2.00E-132	****
TET R	14.75%			95.71%			23.15%			93.14%			92.49%			95.47%		
CIP S	21.78%	3.85E-06	****	94.47%	4.05E-05	****	20.07%	1.93E-08	****	86.56%	4.93E-58	****	85.22%	1.88E-38	****	68.16%	2.65E-175	****
CIP R	15.37%			90.46%			28.24%			98.58%			96.42%			97.80%		
CRO S	17.50%	1		91.02%	1		23.09%	1		93.26%	1		91.31%	1		84.57%	1	
CRO R	4.55%			95.45%			4.55%			100.00%			100.00%			100.00%		
CFX S	16.38%	3.51E-06	****	93.46%	0.19578		26.40%	2.86E-52	****	93.03%	2.64E-10	****	91.41%	1.26E-10	****	82.94%	1.40E-29	****
CFX R	7.55%			89.59%			0.82%			99.59%			98.98%			99.18%		
AZI S	17.23%	1		90.30%	2.91E-16	****	27.29%	2.64E-43	****	92.59%	7.37E-03	**	90.61%	0.024492	*	83.28%	7.88E-22	****
AZI R	13.81%			98.11%			7.02%			96.10%			94.32%			95.21%		

Locus	NEIS1667			NEIS2486			NEIS2582			NEIS2615			NEIS2616			NEIS2618		
Funtional annotation	Nf4 phage island			Hypothetical protein			Hypothetical protein			(Putative) phage associate			(Putative) phage associate			(Putative) phage associate		
	prevalence	adj.p-value	sign.	prevalence	adj.p-value	sign.	prevalence	adj.p-value	sign.	prevalence	adj.p-value	sign.	prevalence	adj.p-value	sign.	prevalence	adj.p-value	sign.
PEN S	26.50%	2.11E-05	****	94.02%	0.01521	***	67.52%	1.25E-44	****	41.03%	4.06E-07	****	78.63%	0.0003	***	72.65%	2.74E-09	****
PEN I	10.13%			92.67%			19.51%			66.35%			81.65%			61.60%		
PEN R	8.29%			88.27%			9.03%			70.11%			88.18%			50.09%		
TET S	15.37%	3.68E-06	****	93.15%	1		38.50%	1.34E-64	****	53.75%	8.27E-16	****	72.09%	6.97E-21	****	61.11%	1	
TET R	8.21%			91.46%			9.61%			70.98%			87.96%			57.16%		
CIP S	6.72%	9.59E-29	****	94.76%	8.11E-14	****	34.60%	2.91E-173	****	65.68%	0.12402		85.41%	5.71E-12	****	63.78%	3.14E-08	****
CIP R	18.01%			88.00%			3.41%			61.05%			76.55%			54.49%		
CRO S	14.56%	1		91.78%	1		16.52%	1		63.21%	1		79.66%	1		59.14%	1	
CRO R	9.09%			90.91%			4.55%			63.64%			81.82%			40.91%		
CFX S	12.69%	1		91.36%	1.02E-09	****	18.91%	6.87E-34	****	65.48%	0.8268		81.54%	0.47		63.76%	1	
CFX R	16.53%			98.78%			0.82%			59.59%			86.53%			62.04%		
AZI S	15.96%	5.14E-29	****	90.36%	2.96E-07	****	19.42%	1.01E-35	****	57.88%	1.64E-33	****	76.06%	3.71E-44	****	58.11%	1	
AZI R	3.01%			96.10%			3.79%			79.62%			95.10%			59.91%		

Locus	NEIS2619			NEIS2621			NEIS2640			NEIS2643			NEIS2646			NEIS2647		
Funtional annotation	(Putative) phage associate			(Putative) phage associate			(Putative) phage associate			(Putative) phage associate			TonB dependent receptor			TonB dependent receptor		
	prevalence	adj.p-value	sign.	prevalence	adj.p-value	sign.	prevalence	adj.p-value	sign.	prevalence	adj.p-value	sign.	prevalence	adj.p-value	sign.	prevalence	adj.p-value	sign.
PEN S	64.10%	8.27E-09	****	13.68%	1.63E-10	****	32.48%	0.029	*	75.21%	0.11388		7.69%	0.00874	**	8.55%	7.02E-04	***
PEN I	61.87%			3.06%			23.26%			67.30%			1.95%			2.27%		
PEN R	49.35%			0.65%			18.72%			73.18%			3.91%			4.93%		
TET S	63.82%	9.75E-04	****	4.91%	5.62E-04	****	31.52%	2.07E-10	****	65.37%	0.07605		5.81%	1.19E-05	****	6.20%	1.73E-04	***
TET R	54.74%			1.73%			18.95%			71.77%			1.87%			2.38%		
CIP S	61.30%	0.01326	*	4.91%	2.54E-10	****	22.64%	1		67.21%	1.39E-06	****	4.77%	7.62E-08	****	5.43%	3.09E-08	****
CIP R	55.70%			1.34%			22.71%			74.91%			1.60%			1.94%		
CRO S	58.01%	1		2.73%	1		22.27%	1		71.12%	0.0975		2.92%	1		3.20%	1	
CRO R	36.36%			9.09%			13.64%			100.00%			4.55%			13.64%		
CFX S	62.51%	1.47E-32	****	3.05%	0.16		22.15%	0.58		74.10%	0.19		3.25%	0.09		3.62%	0.24882	
CFX R	33.47%			0.82%			16.94%			80.41%			0.82%			1.22%		
AZI S	57.68%	4.27E-05	****	3.02%	1		26.11%	1.24E-17	****	70.45%	0.43602		3.47%	3.50E-08	****	3.95%	5.79E-10	****
AZI R	48.33%			2.12%			12.47%			75.17%			0.22%			0.22%		

Locus	NEIS2666			NEIS2691			NEIS2692			NEIS2693			NEIS2705			NEIS2713		
**Funtional annotation**	**Hypothetical protein**			**Methyltransferase**			**Methyltransferase**			**Nf4 phage island**			**Hypothetical protein**			**(Putative) phage associate**		
	**prevalence**	**adj.p-value**	**sign.**	**prevalence**	**adj.p-value**	**sign.**	**prevalence**	**adj.p-value**	**sign.**	**prevalence**	**adj.p-value**	**sign.**	**prevalence**	**adj.p-value**	**sign.**	**prevalence**	**adj.p-value**	**sign.**
**PEN S**	56.41%	8.89E-19	****	43.59%	1.90E-45	****	43.59%	1.16E-44	****	88.89%	2.64E-19	****	70.09%	9.91E-59	****	86.32%	2.00E-79	****
**PEN I**	84.92%			91.61%			91.56%			89.19%			60.34%			25.63%		
**PEN R**	91.34%			96.28%			96.09%			75.61%			30.17%			8.66%		
**TET S**	79.33%	8.35E-11	****	81.78%	9.98E-26	****	81.78%	2.60E-25	****	94.06%	9.75E-20	****	58.01%	1.79E-06	****	50.52%	1.62E-106	****
**TET R**	89.78%			95.29%			95.19%			80.45%			46.24%			10.64%		
**CIP S**	75.93%	3.77E-97	****	80.89%	9.98E-104	****	80.79%	1.93E-102	****	91.37%	5.47E-42	****	72.40%	6.60E-202	****	42.14%	4.14E-207	****
**CIP R**	96.63%			98.92%			98.83%			76.08%			27.29%			4.92%		
**CRO S**	88.17%	1		91.04%	1		90.96%	1		84.33%	1		46.41%	0.025974	*	20.83%	1	
**CRO R**	95.45%			100.00%			100.00%			77.27%			9.09%			4.55%		
**CFX S**	87.04%	2.93E-26	****	89.91%	2.02E-14	****	89.84%	1.27E-14	****	86.11%	7.80E-24	****	50.20%	9.67E-64	****	22.85%	3.99E-38	****
**CFX R**	100.00%			99.18%			99.18%			65.92%			11.84%			1.63%		
**AZI S**	85.93%	6.19E-16	****	89.26%	5.12E-29	****	89.23%	4.59E-28	****	83.03%	1		44.88%	8.58E-05	****	24.08%	1.30E-53	****
**AZI R**	95.55%			99.33%			99.22%			81.29%			54.01%			3.56%		

Locus	NEIS2714			NEIS2718			NEIS2744			NEIS2859			NEIS3177			NEIS3178		
Funtional annotation	(Putative) phage associatec			(Putative) phage associatec			TonB dependent receptor			Hypothetical protein			Phage island X			Hypothetical protein		
	prevalence	adj.p-value	sign.	prevalence	adj.p-value	sign.	prevalence	adj.p-value	sign.	prevalence	adj.p-value	sign.	prevalence	adj.p-value	sign.	prevalence	adj.p-value	sign.
PEN S	64.96%	4.08E-04	***	71.79%	1.15E-05	****	88.89%	1		23.08%	1		6.84%	5.82E-07	****	21.37%	1.29E-21	****
PEN I	56.86%			59.28%			94.78%			16.03%			0.53%			43.25%		
PEN R	65.83%			68.90%			93.11%			14.71%			0.00%			25.79%		
TET S	61.24%	1		55.94%	3.27E-05	****	90.57%	3.66E-04	***	14.73%	1		1.55%	1.77E-03	**	30.88%	5.82E-03	**
TET R	59.26%			66.26%			95.33%			16.43%			0.14%			38.92%		
CIP S	58.77%	1		60.92%	4.24E-11	****	92.61%	1.61E-05	****	19.11%	1		1.43%	1.43E-08	****	40.42%	2.70E-07	****
CIP R	59.33%			71.20%			96.20%			19.17%			0.00%			31.87%		
CRO S	58.34%	1		65.69%	0.9282		94.99%	1		18.69%	1		0.66%	1		37.96%	1	
CRO R	68.18%			90.91%			86.36%			9.09%			0.00%			22.73%		
CFX S	56.64%	1.71E-12	****	68.91%	1		94.23%	0.2262		24.08%	9.75E-12	****	0.72%	1		40.83%	3.01E-03	**
CFX R	74.29%			73.27%			97.35%			10.20%			0.00%			31.22%		
AZI S	59.37%	0.21528		66.20%	1		94.02%	0.0194	*	20.98%	3.89E-06	****	0.76%	0.25194		32.08%	4.00E-34	****
AZI R	53.79%			70.27%			96.99%			13.14%			0.00%			55.01%		

Locus	NEIS3179			NEIS3180			NEIS3181			NEIS3182			NEIS3183			NEIS3184		
Funtional annotation	Hypothetical protein			Hypothetical protein			(Putative) phage associate			Methyltransferase			Hypothetical protein			DNA transcription		
	prevalence	adj.p-value	sign.	prevalence	adj.p-value	sign.	prevalence	adj.p-value	sign.	prevalence	adj.p-value	sign.	prevalence	adj.p-value	sign.	prevalence	adj.p-value	sign.
**PEN S**	98.29%	2.29E-03	****	92.31%	6.04E-81	****	86.32%	0.01092	*	73.50%	3.12E-04	***	93.16%	1		37.61%	1.65E-16	****
**PEN I**	93.57%			68.62%			83.23%			57.44%			96.47%			67.88%		
**PEN R**	89.76%			95.81%			88.83%			51.68%			96.65%			76.82%		
**TET S**	94.32%	1		77.65%	1		74.03%	1.61E-19	****	53.10%	1		96.51%	1		57.11%	3.13E-21	****
**TET R**	92.11%			78.81%			88.94%			56.00%			96.78%			76.34%		
**CIP S**	93.23%	1		63.54%	4.30E-171	****	86.08%	0.00858	**	65.54%	7.39E-33	****	93.85%	1		61.49%	6.48E-20	****
**CIP R**	91.54%			95.64%			89.90%			47.19%			95.21%			74.91%		
**CRO S**	91.68%	1		80.85%	1		88.68%	1		58.05%	1		93.22%	1		70.11%	1	
**CRO R**	100.00%			90.91%			90.91%			81.82%			100.00%			68.18%		
**CFX S**	95.05%	1		78.30%	5.39E-39	****	86.84%	1.77E-06	****	58.92%	1.14E-07	****	96.08%	1		68.73%	3.63E-14	****
**CFX R**	92.65%			98.98%			94.90%			72.86%			94.29%			85.51%		
**AZI S**	90.98%	1		84.47%	4.42E-54	****	86.44%	5.58E-05	****	50.21%	1.76E-50	****	92.08%	1.32E-10	****	68.14%	5.35E-15	****
**AZI R**	92.65%			59.13%			92.32%			77.73%			98.00%			81.85%		

Locus	NEIS3186			NEIS3188			NEIS3189			NEIS3193			NEIS3195			NEIS3196		
Funtional annotation	Phage island X			Toxin-antitoxin system			TspB virulence factor			Hypothetical protein			Phage island X			Hypothetical protein		
	prevalence	adj.p-value	sign.	prevalence	adj.p-value	sign.	prevalence	adj.p-value	sign.	prevalence	adj.p-value	sign.	prevalence	adj.p-value	sign.	prevalence	adj.p-value	sign.
PEN S	5.98%	3.88E-06	****	18.80%	9.20E-119	****	98.29%	0.012168	*	86.32%	2.92E-43	****	6.84%	5.82E-07	****	86.32%	6.06E-06	****
PEN I	0.42%			24.74%			96.94%			89.40%			0.53%			89.87%		
PEN R	0.00%			67.50%			93.85%			67.97%			0.00%			95.25%		
TET S	1.29%	4.95E-03	**	16.93%	3.88E-54	****	98.84%	1.47E-05	****	86.56%	4.30E-03	**	1.55%	1.77E-03	**	88.89%	0.019734	*
TET R	0.09%			48.02%			94.91%			80.12%			0.14%			93.19%		
CIP S	1.29%	1.36E-07	****	9.72%	1.22E-285	****	96.04%	3.36E-05	****	92.04%	6.31E-75	****	1.43%	1.43E-08	****	87.89%	3.28E-18	****
CIP R	0.00%			59.93%			92.49%			70.68%			0.00%			95.42%		
CRO S	0.62%	1		35.62%	0.011076	*	94.04%	1		82.21%	1		0.66%	1		92.67%	1	
CRO R	0.00%			77.27%			95.45%			95.45%			0.00%			86.36%		
CFX S	0.65%	1		32.69%	1.44E-59	****	96.08%	7.43E-09	****	80.67%	1.54E-33	****	0.72%	1		92.66%	1	
CFX R	0.00%			71.63%			88.57%			98.98%			0.00%			91.22%		
AZI S	0.68%	0.65442		36.20%	4.20E-04	****	93.71%	1		78.29%	7.53E-36	****	0.76%	0.25194		91.65%	2.64E-08	****
AZI R	0.00%			44.54%			94.21%			94.99%			0.00%			97.22%		

Locus	NEIS3197			NEIS3198			NEIS3200			NEIS3202			NEIS3203			NEIS3204		
Funtional annotation	Hypothetical protein			Hypothetical protein			Hypothetical protein			DNA replication			(Putative) phage associate			Phage island X		
	prevalence	adj.p-value	sign.	prevalence	adj.p-value	sign.	prevalence	adj.p-value	sign.	prevalence	adj.p-value	sign.	prevalence	adj.p-value	sign.	prevalence	adj.p-value	sign.
**PEN S**	85.47%	1.45E-18	****	81.20%	1		55.56%	9.59E-07	****	3.42%	4.56E-03	**	2.56%	2.66E-03	**	7.69%	4.36E-08	****
**PEN I**	96.99%			86.39%			29.85%			5.49%			6.43%			0.53%		
**PEN R**	88.73%			88.45%			27.47%			2.23%			2.89%			0.00%		
**TET S**	97.03%	2.04E-04	***	88.37%	1		45.48%	4.21E-27	****	9.30%	3.61E-10	****	10.34%	1.44E-09	****	1.68%	5.69E-04	***
**TET R**	92.44%			88.61%			23.57%			2.80%			3.55%			0.14%		
**CIP S**	97.62%	9.59E-21	****	75.50%	8.66E-13	****	37.23%	5.80E-20	****	3.72%	0.60762		3.96%	1		1.48%	6.72E-09	****
**CIP R**	90.89%			84.76%			23.92%			2.33%			3.15%			0.00%		
**CRO S**	93.98%	1		78.37%	1		31.37%	1		2.71%	1		3.20%	1		0.68%	1	
**CRO R**	100.00%			81.82%			22.73%			0.00%			0.00%			0.00%		
**CFX S**	92.48%	3.03E-14	****	83.02%	1		32.17%	3.93E-10	****	3.27%	4.70E-04	***	3.87%	4.24E-05	****	0.75%	1	
**CFX R**	100.00%			82.45%			17.55%			0.20%			0.20%			0.00%		
**AZI S**	93.40%	1.16E-09	****	80.32%	2.11E-03	**	32.03%	5.59E-13	****	3.27%	1	*	3.95%	0.08346		0.79%	0.25818	
**AZI R**	98.55%			86.30%			19.15%			1.78%			1.78%			0.00%		

Locus	NEIS3205			NEIS3206			NEIS3207			NEIS3208			NEIS3209			NEIS3210		
Funtional annotation	(Putative) phage associatec			Hypothetical protein			Phage island X			Hypothetical protein			Hypothetical protein			Hypothetical protein		
	prevalence	adj.p-value	sign.	prevalence	adj.p-value	sign.	prevalence	adj.p-value	sign.	prevalence	adj.p-value	sign.	prevalence	adj.p-value	sign.	prevalence	adj.p-value	sign.
PEN S	3.42%	1.37E-05	****	7.69%	1.02E-06	****	8.55%	2.54E-09	****	64.96%	2.94E-03	**	18.80%	6.66E-18	****	57.26%	3.07E-13	****
PEN I	7.01%			31.28%			0.53%			68.88%			62.39%			28.27%		
PEN R	2.51%			28.68%			0.00%			60.71%			60.24%			21.51%		
TET S	11.37%	3.43E-12	****	36.82%	3.81E-04	***	1.81%	1.80E-04	***	66.28%	1		43.67%	1.21E-25	****	40.44%	1.28E-19	****
TET R	3.55%			27.90%			0.14%			66.17%			66.17%			22.21%		
CIP S	4.67%	0.14118		26.26%	4.34E-05	****	1.53%	3.17E-09	****	70.97%	0.09126		54.81%	1.83E-12	****	33.51%	8.74E-25	****
CIP R	2.85%			33.16%			0.00%			66.41%			66.06%			19.34%		
CRO S	3.37%	1		29.05%	0.053664		0.70%	1		67.73%	0.8502		60.17%	1		23.60%	1	
CRO R	0.00%			0.00%			0.00%			40.91%			36.36%			9.09%		
CFX S	4.07%	1.12E-05	****	30.14%	1.20E-52	****	0.77%	1		72.85%	1.88E-94	****	63.66%	1.88E-61	****	26.17%	2.11E-38	****
CFX R	0.20%			2.24%			0.00%			24.49%			24.08%			3.06%		
AZI S	4.14%	0.076752		34.76%	2.09E-44	****	0.82%	0.15834		66.48%	4.60E-04	***	58.61%	5.94E-16	****	26.73%	1.29E-08	****
AZI R	1.89%			11.80%			0.00%			74.28%			73.94%			16.70%		

Locus	NEIS3211			NEIS3213			NEIS3214			NEIS3215			NEIS3216			NEIS3217		
Funtional annotation	DNA transcription			Hypothetical protein			(Putative) phage associate			Hypothetical protein			Hypothetical protein			Hypothetical protein		
	prevalence	adj.p-value	sign.	prevalence	adj.p-value	sign.	prevalence	adj.p-value	sign.	prevalence	adj.p-value	sign.	prevalence	adj.p-value	sign.	prevalence	adj.p-value	sign.
**PEN S**	13.68%	2.25E-31	****	4.27%	6.85E-05	****	86.32%	5.06E-70	****	84.62%	1.79E-08	****	18.80%	1.63E-20	****	8.55%	2.53E-05	****
**PEN I**	41.19%			2.06%			21.04%			86.76%			8.54%			1.00%		
**PEN R**	21.32%			0.19%			9.50%			94.04%			21.51%			0.56%		
**TET S**	25.32%	1.97E-06	****	4.26%	4.17E-09	****	39.66%	6.97E-61	****	86.30%	6.68E-04	***	8.91%	3.35E-04	***	1.68%	1	
**TET R**	36.21%			0.56%			10.92%			91.97%			15.40%			0.89%		
**CIP S**	39.85%	1.73E-24	****	3.57%	2.29E-23	****	36.18%	8.50E-154	****	84.13%	4.98E-21	****	5.20%	2.11E-51	****	2.24%	1.41E-05	****
**CIP R**	24.91%			0.00%			5.31%			93.39%			20.29%			0.47%		
**CRO S**	30.98%	1		1.40%	1		18.32%	1		89.93%	1		13.37%	1		1.15%	1	
**CRO R**	13.64%			0.00%			0.00%			81.82%			18.18%			0.00%		
**CFX S**	33.02%	5.23E-24	****	1.62%	0.0702		20.60%	6.65E-38	****	90.83%	1		15.11%	1.58E-04	***	1.35%	0.2652	
**CFX R**	11.43%			0.00%			0.82%			89.80%			7.55%			0.00%		
**AZI S**	25.88%	3.34E-56	****	1.86%	0.31902		20.92%	3.19E-45	****	88.86%	9.36E-11	****	14.77%	1.69E-03	**	1.30%	4.01E-03	**
**AZI R**	54.68%			0.56%			3.12%			96.10%			9.47%			0.00%		

Locus	NEIS3218			NEIS3219			NEIS3220			NEIS3221			NEIS3222			NEIS3223		
**Funtional annotation**	**(Putative) phage associate**			**Hypothetical protein**			**DNA transcription**			**(Putative) phage associate**			**Phage island X**			**Hypothetical protein**		
	**prevalence**	**adj.p-value**	**sign.**	**prevalence**	**adj.p-value**	**sign.**	**prevalence**	**adj.p-value**	**sign.**	**prevalence**	**adj.p-value**	**sign.**	**prevalence**	**adj.p-value**	**sign.**	**prevalence**	**adj.p-value**	**sign.**
**PEN S**	85.47%	4.79E-18	****	2.56%	1.95E-04	***	87.18%	1		3.42%	4.13E-05	****	5.98%	7.23E-06	****	86.32%	1.29E-69	****
**PEN I**	96.99%			6.96%			93.09%			7.49%			0.53%			21.04%		
**PEN R**	88.92%			2.89%			91.62%			2.98%			0.00%			9.59%		
**TET S**	97.03%	2.82E-04	***	11.11%	1.01E-10	****	87.98%	2.03E-04	***	12.14%	1.17E-12	****	1.42%	5.42E-03	**	39.66%	1.14E-60	****
**TET R**	92.53%			3.73%			93.56%			3.92%			0.14%			10.97%		
**CIP S**	97.62%	4.11E-20	****	4.43%	1		93.57%	1		4.91%	0.44382		1.38%	3.02E-08	****	36.18%	3.92E-153	****
**CIP R**	91.02%			3.15%			93.18%			3.24%			0.00%			5.35%		
**CRO S**	93.88%	1		3.41%	1		94.39%	1		3.66%	1		0.64%	1		18.34%	1	
**CRO R**	100.00%			0.00%			100.00%			0.00%			0.00%			0.00%		
**CFX S**	92.46%	1.97E-11	****	4.12%	1.15E-05	****	93.28%	4.40E-07	****	4.42%	3.44E-06	****	0.70%	1		20.63%	6.40E-38	****
**CFX R**	99.59%			0.20%			98.98%			0.20%			0.00%			0.82%		
**AZI S**	93.35%	2.02E-10	****	4.17%	0.11934		92.44%	3.14E-05	****	4.45%	0.1248		0.73%	0.41106		20.92%	1.81E-44	****
**AZI R**	98.66%			2.00%			96.88%			2.23%			0.00%			3.23%		

Locus	NEIS3224			NEIS3226			NEIS3228			NEIS3232			NEIS3233			NEIS3234		
Funtional annotation	Phage island X			Membrane protein			Hypothetical protein			Toxin-antitoxin system			Hypothetical protein			Hypothetical protein		
	prevalence	adj.p-value	sign.	prevalence	adj.p-value	sign.	prevalence	adj.p-value	sign.	prevalence	adj.p-value	sign.	prevalence	adj.p-value	sign.	prevalence	adj.p-value	sign.
**PEN S**	8.55%	2.85E-09	****	0.85%	1		95.73%	0.1794		18.80%	1.78E-118	****	85.47%	4.79E-18	****	98.29%	0.9048	
**PEN I**	0.58%			0.74%			95.62%			24.79%			96.99%			99.89%		
**PEN R**	0.00%			0.37%			97.95%			67.50%			88.92%			99.72%		
**TET S**	1.81%	1.80E-04	***	1.42%	0.037674	*	96.51%	0.8268		16.93%	3.44E-54	****	97.03%	2.80E-04	***	99.74%	1	
**TET R**	0.14%			0.23%			98.18%			48.06%			92.58%			99.77%		
**CIP S**	1.57%	1.50E-09	****	1.33%	6.40E-08	****	92.56%	1		9.77%	6.22E-271	****	97.66%	1.02E-20	****	93.66%	1	
**CIP R**	0.00%			0.00%			92.79%			59.93%			90.98%			94.08%		
**CRO S**	0.72%	1		0.60%	1		92.15%	1		35.62%	0.011076	*	94.04%	1		93.28%	1	
**CRO R**	0.00%			0.00%			86.36%			77.27%			100.00%			100.00%		
**CFX S**	0.77%	1		0.67%	1		94.63%	1		32.72%	1.61E-59	****	92.58%	4.76E-14	****	99.83%	1	
**CFX R**	0.00%			0.00%			92.04%			71.63%			100.00%			99.80%		
**AZI S**	0.82%	0.15834		0.65%	0.63492		92.73%	2.29E-07	****	36.23%	4.24E-04	***	93.46%	4.57E-10	****	91.20%	3.26E-20	****
**AZI R**	0.00%			0.00%			97.66%			44.54%			98.66%			99.11%		
